# Semaphorin-3C signals through Neuropilin-1 and PlexinD1 receptors to inhibit pathological angiogenesis

**DOI:** 10.15252/emmm.201404922

**Published:** 2015-07-20

**Authors:** Wan-Jen Yang, Junhao Hu, Akiyoshi Uemura, Fabian Tetzlaff, Hellmut G Augustin, Andreas Fischer

**Affiliations:** 1 Vascular Signaling and Cancer (A270), German Cancer Research Center (DKFZ-ZMBH Alliance)Heidelberg, Germany; 2 Vascular Biology and Tumor Angiogenesis, Medical Faculty Mannheim (CBTM), Heidelberg UniversityMannheim, Germany; 3 Vascular Oncology and Metastasis (A190), German Cancer Research Center (DKFZ-ZMBH Alliance)Heidelberg, Germany; 4 Department of Retinal Vascular Biology, Nagoya City University Graduate School of Medical SciencesNagoya, Japan; 5 Department of Medicine I and Clinical Chemistry, Heidelberg UniversityHeidelberg, Germany

**Keywords:** angiogenesis, semaphorin, retinopathy of prematurity, Sema3C

## Abstract

Retinopathy of prematurity causes visual impairment due to destructive neoangiogenesis after degeneration of the retinal microvasculature. This study was aimed at analyzing whether local delivery of Semaphorin-3C (Sema3C) suppresses pathological retinal angiogenesis. Sema3C exerted potent inhibiting effects in cellular models of angiogenesis. In an endothelial cell xenotransplantation assay, Sema3C acted primarily on immature microvessels by inducing endothelial cell apoptosis. Intravitreal administration of recombinant Sema3C disrupted endothelial tip cell formation and cell–cell contacts, which led to decreased vascular bed expansion and vessel branching in the growing retinal vasculature of newborn mice, while not affecting mature vessels in the adult retina. Sema3C administration strongly inhibited the formation of pathological pre-retinal vascular tufts during oxygen-induced retinopathy. Mechanistically, Sema3C signaled through the receptors Neuropilin-1 and PlexinD1, which were strongly expressed on vascular tufts, induced VE-cadherin internalization, and abrogated vascular endothelial growth factor (VEGF)-induced activation of the kinases AKT, FAK, and p38MAPK. This disrupted endothelial cell junctions, focal adhesions, and cytoskeleton assembly resulted in decreased cell migration and survival. Thus, this study identified Sema3C as a potent and selective inhibitor of pathological retinal angiogenesis.

See also: **G Serini & L Tamagnone** (October 2015)

## Introduction

Angiogenesis is a prerequisite for embryonic development. Reactivation of this embryo–fetal program occurs in the adult, for example, during wound healing, tissue growth, or the menstrual cycle. However, excessive or uncontrolled vessel sprouting is seen in a number of diseases, most notably malignant tumors and proliferative eye diseases (Chung & Ferrara, 2011). Several drugs, which interfere with vascular endothelial growth factor (VEGF) signaling, have been approved for the treatment of advanced carcinomas and the wet form of macular degeneration (Barakat & Kaiser, 2009; Welti *et al*, 2013). While these drugs often only slow down tumor growth for a while, they have revolutionised treatment of the wet form of age-related macular degeneration. In this disease, the local application of anti-VEGF compounds prevents further outgrowth and leakage of new, immature blood vessels.

Similar therapeutics would also be highly desirable to treat preterm babies suffering from retinopathy of prematurity (ROP). The human retinal vasculature develops between the 16^th^ week of gestation and birth. Therefore, preterm born babies who receive intensive neonatal care therapy with increased oxygen supply are at high risk of developing ROP, which is the major ocular disorder of the neonate and the dominant cause of visual impairment in childhood. In phase 1 of ROP, relative hyperoxia and decreased VEGF levels cause vessel regression in the retina. Phase 2 of ROP involves relative hypoxia and exaggerated VEGF, Epo, and Angiopoietin-2 secretion from neurons and astrocytes leading to excessive neovascularization, which is often misguided toward the vitreous body and lens. This can cause hemorrhage and retinal detachment leading to vision loss (Sapieha *et al*, 2010). The use of anti-VEGF agents is an emerging treatment for acute ROP, but studies analyzing long-term effects are still rare (Mutlu & Sarici, 2013).

Vascular endothelial growth factor acts through VEGF receptors and Neuropilin receptors on endothelial cells. It induces the formation of tip cells, which are motile and guide new vessel sprouts. In addition to VEGF, the further growth of new vessel sprouts is also regulated by attractive and repulsive cues. This is similar to neurogenesis, where the outgrowth of axons is coordinated by neuronal guidance molecules of the Netrin, Slit, Ephrin, and Semaphorin families (Larrivee *et al*, 2009; Adams & Eichmann, 2010).

Class-3 Semaphorins (Sema3A to Sema3G) are secreted proteins that were originally identified as factors controlling axonal branching and pathfinding in the nervous system. They signal mainly through a receptor complex in which a Neuropilin (Nrp-1 or Nrp-2) associates with a Plexin receptor. The Plexin receptors modulate activity of GTPases, which cause changes in the cytoskeletal assembly and cell adhesive forces (Kruger *et al*, 2005; Negishi *et al*, 2005).

Among Sema3 proteins, Sema3C is still a poorly understood molecule. It is expressed during development in neurons, neural crest cells, myocardium, and kidney (Feiner *et al*, 2001; Niquille *et al*, 2009; Reidy & Tufro, 2011). Sema3C-deficient mice show a defective cardiac outflow tract (Feiner *et al*, 2001), and decreased ureteric bud branching (Reidy & Tufro, 2011). The cardiac phenotype is partially phenocopied by loss of PlexinD1 expression in endothelial cells (Gitler *et al*, 2004), suggesting an essential role of paracrine Sema3C signaling during cardiogenesis.

The function of class-3 Semaphorin proteins is regulated by furin or furin-like endoproteinases. These enzymes can recognize two or three consensus RXK/RR motifs in the Plexin-Sema-Ig (PSI) domain and C-terminal basic domain (Adams *et al*, 1997). Metalloproteinases also cleave Sema3C at the C-terminus, and it was assumed that this cleavage of the last 13 amino acids releases Sema3C from the extracellular matrix (Esselens *et al*, 2010). The consequence of cleavage within the PSI domain that significantly shortens the protein (Sema3Cp60 isoform) is not fully understood, but the repulsive activity of Sema3C during the outgrowth of sympathetic axons is increased when this processing is blocked (Adams *et al*, 1997).

Sema3C is not expressed by human endothelial cells (Serini *et al*, 2003), and there are no data indicating a role for physiological angiogenesis. However, Sema3C expression is high in gastric and lung carcinoma as well as glioma (Martin-Satue & Blanco, 1999; Rieger *et al*, 2003; Miyato *et al*, 2012). These findings together with the known anti-angiogenic functions of Sema3A and Sema3F (Gu & Giraudo, 2013) indicate a potential role of Sema3C on blood vessels under pathological conditions. This study was aimed at elucidating how Sema3C regulates angiogenesis and exploring its potential for anti-angiogenic therapy.

## Results

### Sema3C inhibits endothelial tube formation and sprouting angiogenesis

**Figure 1:**
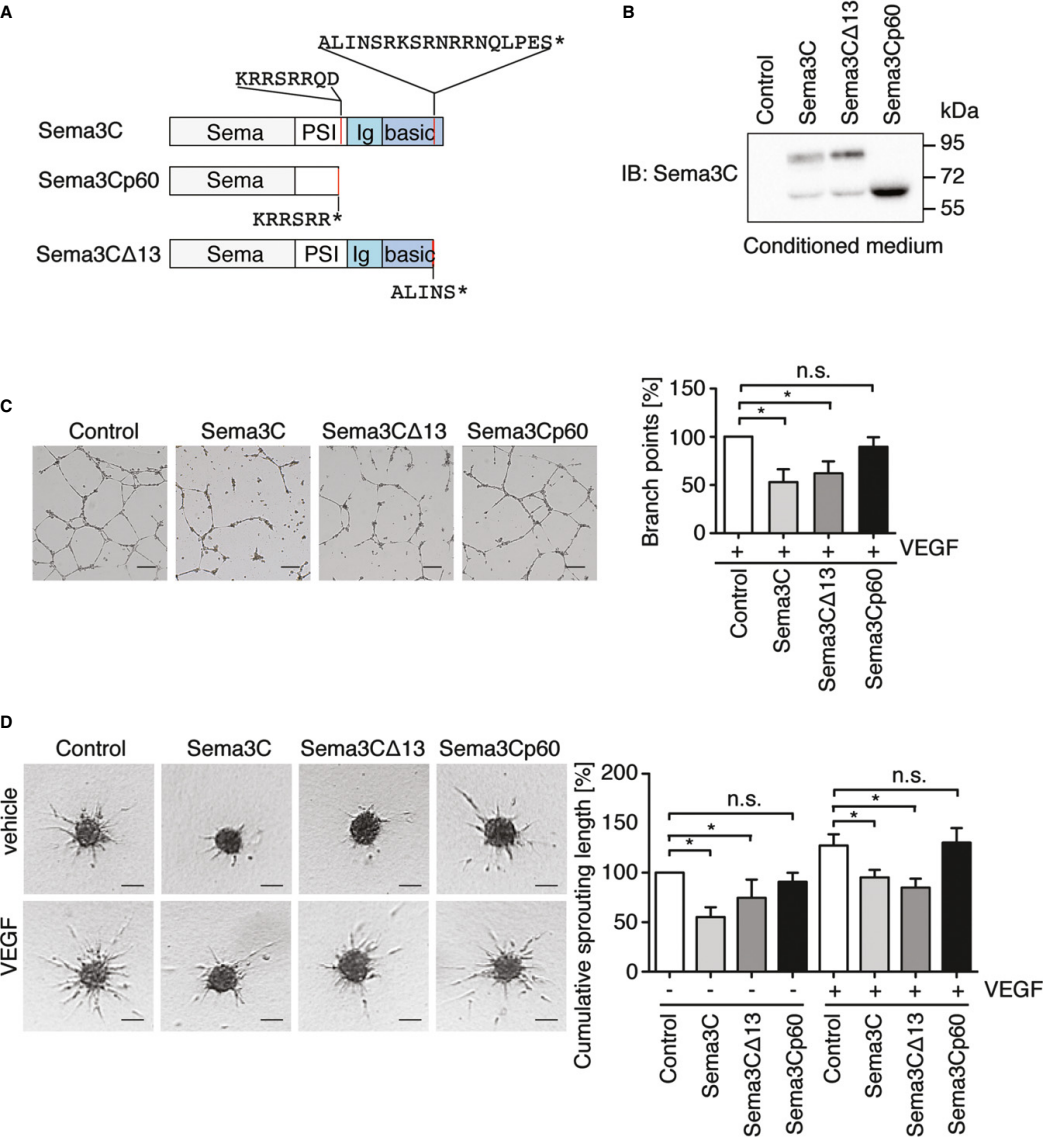
Sema3C inhibits endothelial tube formation and sprouting angiogenesis Sema3C domain structure with two consensus motifs for furin and furin-like proteases. Sema3CΔ13 and Sema3Cp60 expression vectors were generated by replacing the amino acids in the consensus motifs with stop codons (asterisk).
Conditioned medium was collected from pericytes 48 h after adenoviral transduction. Western blotting verified the secretion of Sema3CΔ13 (81 kDa) and Sema3Cp60 (60 kDa) isoforms.
HUVECs were suspended in conditioned medium with VEGF (25 ng/ml) and plated on Matrigel. Branch points of the capillary-like network were counted after 18 h. *n* = 4 independent experiments; scale bar, 200 μm. Control vs. Sema3C, *P =* *0.0375 and control vs. Sema3Δ13, *P =* *0.0162.
HUVEC spheroids in collagen were treated with conditioned medium with or without VEGF (25 ng/ml). After 24 h, the cumulative sprout length per spheroid was quantified. Data are representative of three independent experiments with ten spheroids per group. Scale bar, 100 μm. Control vs. Sema3C (− VEGF), *P =* *0.0276; control vs. Sema3CΔ13 (− VEGF), *P =* *0.0443; control vs. Sema3C (+ VEGF), *P =* *0.028 and control vs. Sema3CΔ13 (+ VEGF), *P =* *0.032. Data information: (C, D) Mean ± s.e.m; n.s., non-significant using paired Student's *t-*test.

We first evaluated Sema3C expression in different human primary endothelial cells and mural cells. We found that endothelial cells freshly isolated from the vein of umbilical cord (HUVEC) and human brain microvascular endothelial cells express very little Sema3C mRNA. On the contrary, mural cells in culture express higher levels of Sema3C mRNA and protein (Supplementary Fig S1A and B). Based on these finding, we used brain-derived pericytes as producer cells for Sema3C-conditioned medium. We generated adenoviral expression vectors for full-length Sema3C and the two isoforms: the truncated Sema3CΔ13 that lacked the last 13 amino acids in the C-terminal basic domain and corresponds to metalloproteinase-dependent cleavage (Esselens *et al*, 2010), and Sema3Cp60 that resembles furin cleavage within the PSI domain (Fig 1A). These proteins were expressed in pericytes (Supplementary Fig S1C). Forced expression of Sema3C and Sema3CΔ13 cDNA led to secretion of proteins with the same molecular weight (81 kDa) (Fig 1B). Sema3C was further cleaved into the p60 isoform and this could be prevented by the furin convertase inhibitor Decanoyl-RVKR-CMK but not by the metalloproteinase inhibitor Batimastat (Supplementary Fig S1D).

To assess the effect of Sema3C proteins in angiogenesis, HUVECs were treated with freshly prepared conditioned medium. VEGF induced the formation of a tube-like HUVEC network on Matrigel. The treatment with Sema3C- and Sema3CΔ13-conditioned medium strongly inhibited endothelial tube formation. However, Sema3Cp60 had no effect (Fig 1C).

Next, we validated these findings in a three-dimensional angiogenesis assay, which better resembles the process of endothelial sprouting. VEGF, VEGF-C, and FGF2 stimulated the outgrowth of capillary-like structures, while Sema3C- or Sema3CΔ13-conditioned medium significantly impaired endothelial sprouting and tube elongation (Fig 1D and Supplementary Fig S1E). HUVEC spheroids treated with Sema3Cp60 showed unaltered sprouting (Fig 1D). This indicated that cleavage within the PSI domain abrogated Sema3C functions.

In order to rule out the possibility that other factors within the conditioned media may also be responsible for the observed effects, we employed a purified, recombinant human Sema3C-Fc fusion protein (Supplementary Fig S1F). This protein carried point mutations that prevent cleavage within the PSI domain and has a deletion of C-terminal basic amino acid residues. Application of recombinant Sema3C-Fc, but not IgG-Fc, to HUVEC spheroids similarly inhibited sprouting angiogenesis (Supplementary Fig S1F). Thus, the data indicated that Sema3C acted as a potent anti-angiogenic protein *in vitro*.

### Sema3C inhibits vessel formation of transplanted endothelial cells in mice

**Figure 2:**
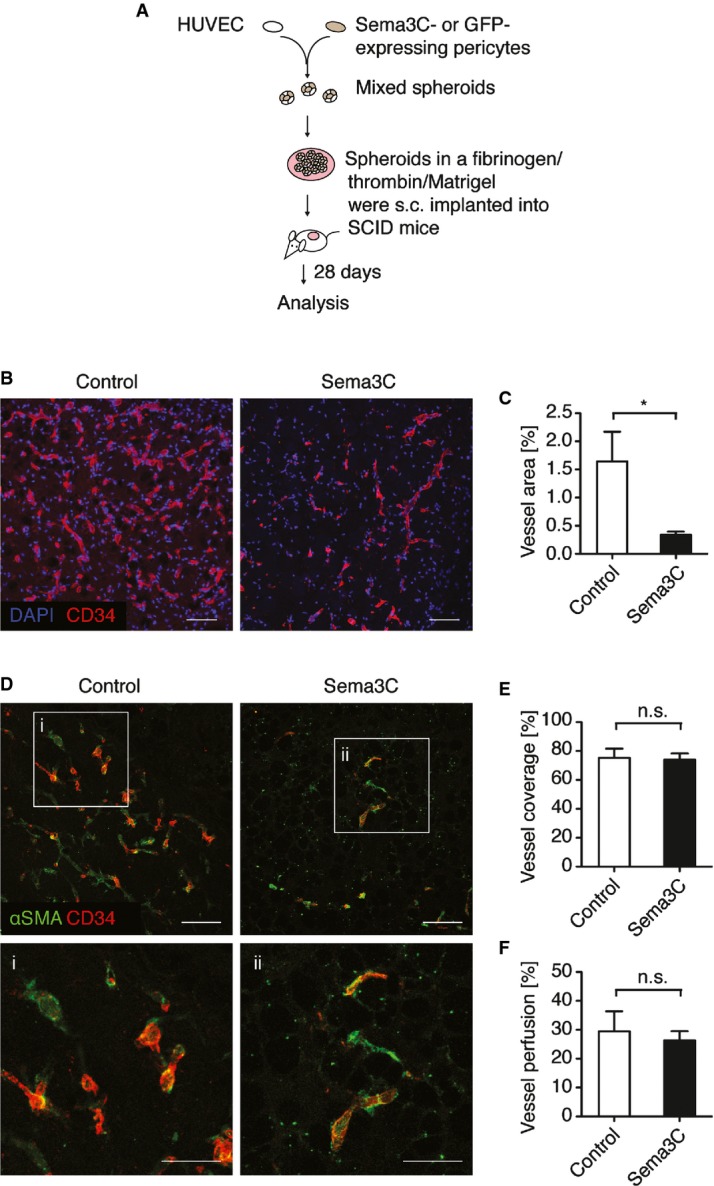
Sema3C inhibits vessel formation in a xenotransplantation model Scheme of the experimental procedure. Pericytes were lentivirally transduced with Sema3C or GFP expression vectors. HUVEC/pericyte spheroids (1:1 ratio) were injected s.c. into SCID mice.
Microvessels within the plugs stained against human-specific CD34. Cell nuclei were stained with DAPI. Scale bar, 100 μm.
The area of CD34-positive microvessels was quantified and normalized to the total area of plug. Results represent the means ± s.e.m of 5 plugs (control) and 7 plugs (Sema3C), *P =* *0.018.
Vessel coverage was assessed by αSMA staining. Scale bar, 100 μm.
Vessel coverage was quantified as the ratio of endothelial cell-associated mural cells to total amount of vessels (*n* = 5 (control) and *n* = 7 (Sema3C)).
Vessel perfusion was evaluated by i.v. injection of FITC-lectin (0.15 mg) 30 min before mice were sacrificed. The data show the percentage of perfused vessels in the plug and normalized to total vessel area (*n* = 5 (control) and *n* = 7 (Sema3C)). Data information: (C, E, F) Mean ± s.e.m; n.s., non-significant using unpaired Student's *t-*test.

We next examined whether Sema3C could also inhibit *de novo* vessel formation *in vivo*. We applied an established model using HUVEC spheroids transplanted within a growth factor-rich matrix into nude mice (Alajati *et al*, 2008; Wustehube *et al*, 2010). We modified the established protocol to co-graft HUVEC and human brain-derived pericytes (Fig 2A). The pericytes were stably transduced with lentiviral expression vectors for Sema3C or GFP as control. The plugs were dissected after 4 weeks, and HUVEC-derived vessels were stained with an antibody that specifically recognizes human CD34. The data showed that pericyte-secreted Sema3C strongly inhibited vessel formation (Fig 2B and C). However, the remaining vessels were well covered by αSMA-positive mural cells (Fig 2D and E) and perfused to a similar extent as those in the control group (Fig 2F). This experiment demonstrated that Sema3C was able to inhibit *de novo* vessel formation *in vivo*.

### Sema3C impairs postnatal retinal angiogenesis

In contrast to humans, mice are born with an avascular retina. Retinal vessels grow postnatal from the central optic disk radially to the periphery following a VEGF gradient. The primary superficial vascular network is established within 7 days, and then the established vascular plexus undergoes remodeling to complete the vascular network (Stahl *et al*, 2010).

**Figure 3:**
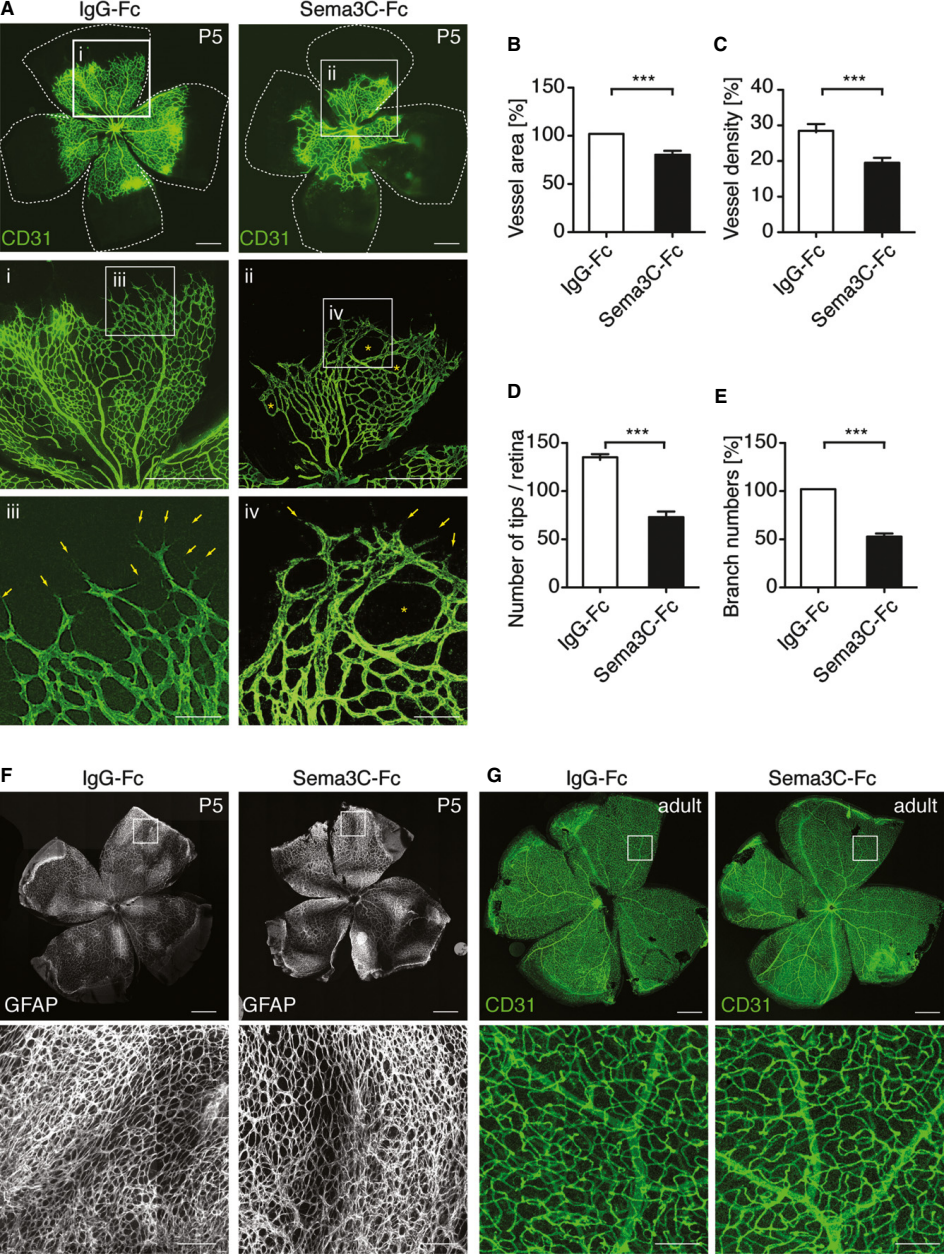
Intravitreal injection of Sema3C inhibits retinal vessel formation A C57BL/6N mice pups at P3 received 1 μg of mouse Sema3C-Fc (right eye) or control IgG-Fc (left eye). The retinal vasculature was examined at P5 by whole-mount CD31 immunofluorescent staining. Representative images show the total retinal vasculature and sprouting tips at the vascular front (iii and iv, arrows) and the enlarged intercapillary space (ii and iv, asterisks). Scale bar, 100 μm and 20 μm (iii and iv).B,C (B) Retinal vessel outgrowth measured as the total area from the central optic disk to the vascular front normalized to control IgG-Fc treatment, ***P =* *0.0009. (C) Vessel density was quantified by CD31-stained vessels and normalized to the total vessel area, ***P =* *0.0002. (Mean ± s.e.m; *n* = 23 retinae in each group from four independent experiments; unpaired Student's *t*-test.) 
D,E (D) Number of sprouting tips per retina, *P < 0.0001. (E) Branch points in the capillary plexus represented as the ratio to the control IgG-treated retinae, ***P <* *0.0001. (Mean ± s.e.m; *n* = 18 retinae in each group from three independent experiments; unpaired Student's *t*-test.) 
F Pups received same treatment as described in (A). The astrocyte layer was examined at P5 by GFAP staining. Scale bar, 100 μm (upper panel) and 20 μm (lower panel). Representative images of six retinae per group.G Six-week-old C57BL/6N male mice were intravitreally injected with Sema3C-Fc (right eye) or control IgG-Fc (left eye), and retinae were collected 2 days later. The retinal vessel network was examined with CD31 immunostaining. Scale bar, 100 μm (upper panel) and 20 μm (lower panel). Representative images of four retinae per group.

To evaluate the effect of Sema3C on blood vessels during sprouting angiogenesis, wild-type C57BL/6N mice at postnatal day P3 were injected with 1 μg recombinant Sema3C or IgG-Fc protein as control in the vitreous humor of the right and left eye, respectively. Two days later, the retinae were collected for analysis. Whole-mount immunostaining of the endothelial cell marker CD31 showed reduced vascularized area and decreased vessel density in Sema3C-treated retinae compared to IgG-Fc treatment (Fig 3A–C). Remarkably, the numbers of sprouting tips at the vascular front were significantly decreased in the Sema3C-treated group (Fig 3Aiv and D), indicating that Sema3C impaired VEGF-driven tip cell formation. Expression of the tip cell-enriched protein ESM1, but not DLL4, was strongly diminished upon Sema3C treatment (Supplementary Fig S2A and B). Also in HUVEC, expression of the tip cell-enriched genes *APLN* and *PDGFB* was decreased upon Sema3C treatment. Sema3C also led to slight induction of the Notch target genes *EFNB2*, *HEY1*, and *DLL4* (Supplementary Fig S2C). Increased Notch activity is well known to inhibit tip cell differentiation (Adams & Eichmann, 2010); therefore, Sema3C appeared to favor stalk cell selection. Consistently, the retinae showed an aberrant vascular network with enlarged intercapillary spaces and fewer vessel branches after Sema3C treatment (Fig 3Aii, Aiv and E). Importantly, the astrocytes that form a template under the vascular bed and that secrete VEGF were not affected by Sema3C (Fig 3F) The relative VEGF-164 mRNA levels in whole retinal explants were not significantly altered between control and Sema3C treatment (1.099 ± 0.5 vs. 1.20 ± 0.37; *n* = 4). As such, Sema3C specifically inhibited the formation of new vascular sprouts during retinal angiogenesis.

In a second experiment, we investigated whether such a short-term treatment would also affect mature blood vessels in the eye of adult mice. We injected recombinant Sema3C protein into the vitreous humor of 6-week-old mice and analyzed the retinae 2 days later. This manipulation did not change the morphology of the fully developed vascular network at all (Fig 3G), indicating that Sema3C acted predominantly on immature blood vessels.

This was further supported by the finding that Sema3C was unable to disrupt a HUVEC monolayer cultured on Xellulin (Supplementary Fig S2D), a hydrogel matrix that allows long-term culture of endothelial cells in a quiescent state.

### Sema3C inhibits adherens junction integrity leading to endothelial cell death

**Figure 4:**
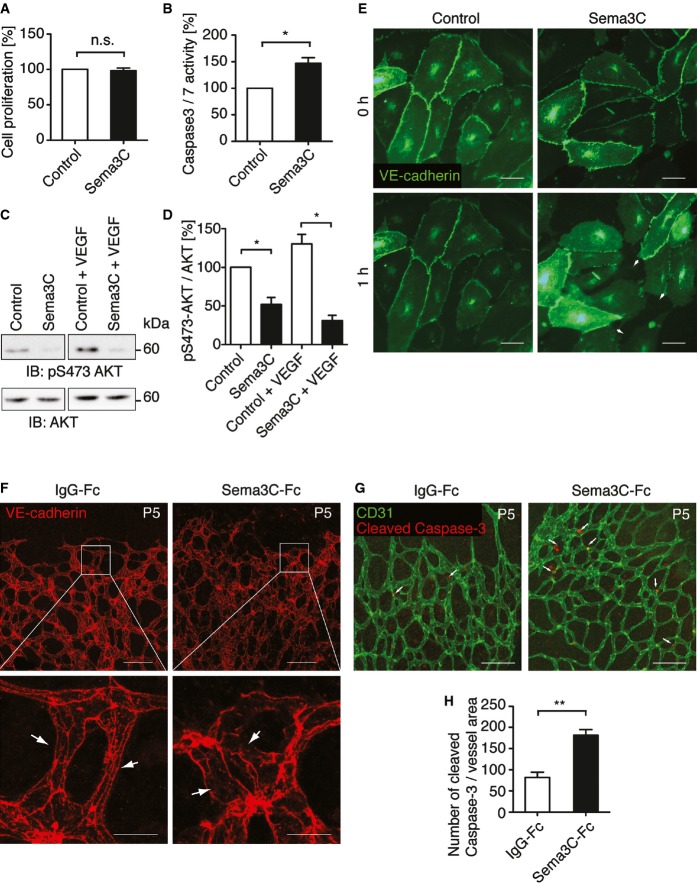
Sema3C inhibits endothelial cell survival and disrupts endothelial cell–cell contacts HUVECs were treated with Sema3C- or GFP-conditioned medium for 24 h. Cell proliferation was quantified by BrdU incorporation (mean ± s.e.m; *n* = 3; n.s., non-significant).
Apoptosis of HUVEC was measured by caspase-3 and caspase-7 activities after 3 h of incubation with Sema3C- or control-conditioned medium (mean ± s.e.m; *n* = 3, *P =* *0.018 using unpaired Student's *t*-test).
Serum-starved HUVECs were incubated with conditioned medium or conditioned medium + VEGF (50 ng/ml) for 15 min. Cell lysates were subjected to SDS–PAGE and immunoblotted with anti-phospho-AKT antibody.
Quantification of phospho-AKT normalized to total AKT levels (mean ± s.e.m; *n* = 4; control vs. Sema3C (− VEGF), *P =* *0.034 and control vs. Sema3C (+ VEGF), *P =* *0.024 using unpaired Student's *t-*test).
HUVECs were adenovirally transduced with VE-cadherin-GFP and plated on ibidi slides (*n* = 4; control vs. Sema3C (− VEGF) at 20× magnification (Supplementary Movies S1 and S2)). Representative pictures are shown at time points 0 and 1 h. Arrowheads indicate gaps between endothelial cells. Scale bar, 100 μm.
C57BL/6N mice pups at P5 received 1 μg of recombinant Sema3C or IgG-Fc proteins. Endothelial cell junctions were examined by whole-mount VE-cadherin immunofluorescent staining 6 h later. Representative images show VE-cadherin at cell junctions (arrows). The linear structure (i) was disrupted in Sema3C-treated retinae (ii). Scale bar, 100 μm (upper panel) and 20 μm (lower panel).
Whole-mount immunostaining of the vascular bed by CD31 and apoptotic cells by cleaved caspase-3 in the retinae at P5 8 h after intravitreal injection of Sema3C or IgG-Fc. Representative images show active caspase-3 signals that co-localize with endothelial cells (arrows). Scale bar, 100 μm.
Quantification of cleaved caspase-3 normalized to area of CD31-stained vessels (mean ± s.e.m; *n* = 5; **P =* *0.007 using paired Student's *t*-test). Source data are available online for this figure.

The observed anti-angiogenic effects of soluble Sema3C in the growing vasculature prompted us to investigate its cellular mechanisms, in particular VEGF-induced endothelial cell proliferation and survival. HUVECs were treated with Sema3C-conditioned medium or control-conditioned medium from GFP-expressing pericytes. Sema3C did not interfere with endothelial cell proliferation as measured by BrdU incorporation into newly synthesized DNA (Fig 4A). Consistently, the activation of extracellular signal-regulated kinase (ERK), which promotes endothelial cell proliferation, was not changed by Sema3C treatment (Supplementary Fig S3A). However, Sema3C impaired endothelial cell survival as evidenced by increased levels of active caspase-3 and caspase-7 in HUVEC (Fig 4B). The elevated endothelial cell apoptosis rate was accompanied by a marked decrease in phosphorylated AKT (Fig 4C and D). AKT acts downstream of VEGF signaling and protects endothelial cells from apoptosis (Gerber *et al*, 1998). Therefore, the data showed that Sema3C mainly suppresses endothelial cell survival but not cell proliferation.

We also observed pronounced changes of endothelial cell morphology upon the addition of Sema3C-conditioned medium. This included the breakdown of adherens junctions with less colocalization of VE-cadherin and β-catenin. This was not observed by treatment with the p60 isoform (Supplementary Fig S3B). Also cell fractionation showed less VE-Cadherin at the cell membrane (Supplementary Fig S3C). To better visualize this, HUVECs were adenovirally transduced with GFP-tagged VE-cadherin and observed by time-lapse microscopy (Fig 4E and Supplementary Movies S1 and S2). VE-cadherin is an endothelial cell-specific adherens junction protein that associates with the VEGF receptor-2 (VEGFR2) at cell junctions. This interaction is essential for VEGF-induced cell survival signaling (Carmeliet *et al*, 1999). Treatment with control-conditioned medium did not alter the localization of VE-cadherin, which was predominantly found at cell–cell contacts. In contrast, Sema3C induced dissociation of VE-cadherin and VEGFR2 from the cell junctions and internalization into the cytosol. Subsequently, this resulted in the formation of intercellular gaps within the endothelial monolayer (Fig 4E and Supplementary Fig S3D).

VE-cadherin is integral to maintain vessel integrity. Intravitreal administration of recombinant Sema3C at postnatal day 5 led to a breakdown of VE-Cadherin at cell junctions as observed 6 h later (Fig 4F). This was accompanied by increased endothelial cell apoptosis (Fig 4G and H). Increased endothelial cell apoptosis after Sema3C delivery to endothelial cells was also observed in the HUVEC/pericyte xenotransplantation experiment in nude mice (Fig 2A). Notably, Sema3C overexpression in pericytes had no influence on pericyte cell death (Supplementary Fig S4A). However, increased rates of cleaved caspase-3 were detected in CD34-positive endothelial cells when co-grafted with Sema3C-secreting pericytes (Supplementary Fig S4B and C).

### Sema3C impairs VEGF-induced endothelial cell migration through inhibiting focal adhesion kinase and p38MAPK signaling

**Figure 5:**
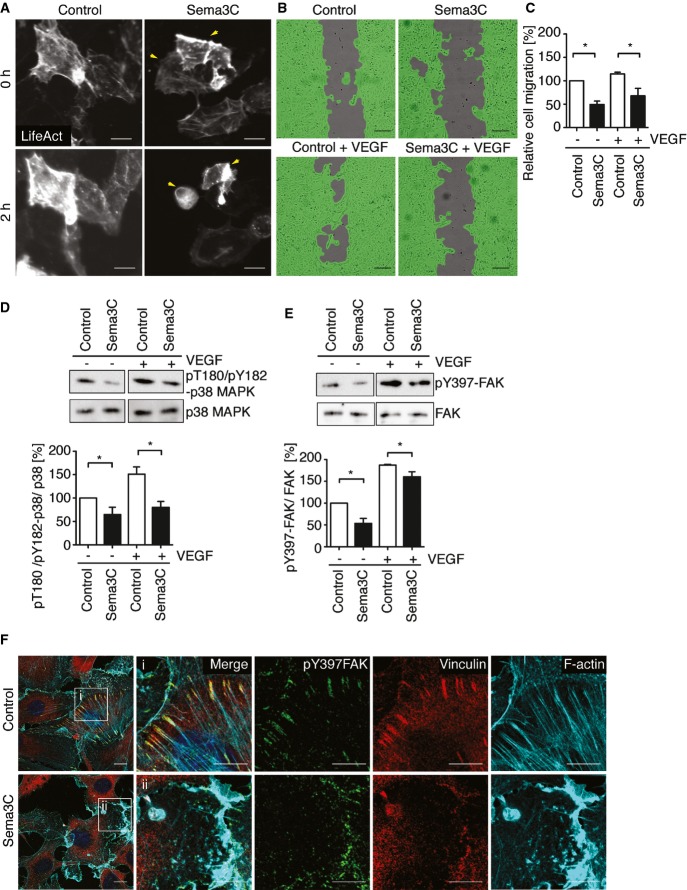
Sema3C suppresses endothelial cell adhesion and migration by inhibiting FAK and p38MAPK signaling pathways A HUVECs were transduced with adenoviral vector expressing RFP-conjugated F-actin binding peptide (LifeAct). Time-lapse imaging of F-actin in HUVEC was obtained at 20× magnification (Supplementary Movies S3 and S4). Representative images are shown at 0 and 2 h. Arrowheads indicate detached cells after Sema3C treatment. Scale bar, 100 μm.B Endothelial cell migration was determined using a scratch wound assay with a co-culture model. A wound was generated in a confluent HUVEC monolayer, and cells were cultured in basal medium with or without VEGF (25 ng/ml) in a 24-well plate. At the same time, the transwells containing Sema3C or GFP-expressing pericytes. Representative pictures show remaining gaps within the HUVEC monolayer. Scale bar, 200 μm.
C Migration distance was measured after 24 h and normalized to control treatment (mean ± s.e.m; *n* = 3 independent experiments, control vs. Sema3C (− VEGF), *P =* *0.021 and control vs. Sema3C (+ VEGF), *P =* *0.039 using unpaired Student's *t-*test).D,E (D) Serum-starved HUVECs were treated with Sema3C- or control-conditioned medium with or without VEGF (50 ng/ml) for 10 min. Cell lysates were subjected to SDS–PAGE and probed with anti-phosphoT180/Y182 p38MAPK antibody. Quantification of p38MAPK activation was normalized to total p38MAPK and presented as the ratio to the cells with control medium without VEGF treatment. Control vs. Sema3C (− VEGF), *P =* *0.046 and control vs. Sema3C (+ VEGF), *P =* *0.043. (E) HUVECs were treated as described before and the activation of FAK detected by anti-phosphoY397 FAK antibody and normalized to the total amount of FAK. Control vs. Sema3C (− VEGF), *P =* *0.033 and control vs. Sema3C (+ VEGF), *P =* *0.047. (Mean ± s.e.m; *n* = 3 independent experiments; unpaired Student's *t*-test.)F Serum-starved HUVECs were treated with conditioned medium for 15 min. Focal adhesion proteins were examined with anti-phosphoY397FAK and anti-vinculin antibodies. F-actin was stained with Alexa Fluor 546-conjugated phalloidin. Merged images show the coupling of stress fibers with focal adhesion complexes in the cells treated with control medium. Inset, phospho-Y397FAK and vinculin formed focal adhesions that were stained in an elongated dot-like structure (i). Lower panel (ii), Sema3C treatment induced F-actin disassembly and membrane ruffling. Phospho-Y397FAK and vinculin were randomly distributed at the peripheral membrane. Scale bars, 10 μm. Source data are available online for this figure.

VE-cadherin associates with endothelial cell migration through adherens junctions, which are linked to the actin cytoskeleton. To assess the effect of Sema3C on actin fibers, HUVECs were transduced with the fluorescent F-actin binding peptide LifeAct-RFP (Riedl *et al*, 2008) and imaged by time-lapse microscopy. Sema3C induced not only the internalization of VE-cadherin, but also the depolymerization of the F-actin bundles. This finally led to endothelial cell detachment from the extracellular matrix (Fig 5A and Supplementary Movies S3 and S4), which causes anoikis.

The findings prompted us to investigate endothelial cell migration since this process relies on dynamic changes of actin filaments and adhesive structures with the extracellular matrix. Sema3C treatment delayed the closure of a defined gap within a HUVEC monolayer, indicating decreased endothelial cell migration. This could not be rescued by the addition of 25 ng/ml VEGF (Fig 5B and C). Additionally, Sema3C treatment impaired cell adhesion and haptotaxis on different matrix proteins and poly-L-lysine (Supplementary Fig S5A and B).

VEGF signaling activates p38MAPK and focal adhesion kinase (FAK), which are both essential for endothelial cell migration (Rousseau *et al*, 1997; Avraham *et al*, 2003). VEGF-mediated phosphorylation of p38MAPK at residues threonine-180 and tyrosine-182 promotes the assembly of actin stress fibers. Sema3C significantly inhibited the activation of p38MAPK in HUVEC (Fig 5D). Sema3C also inhibited phosphorylation of FAK at tyrosine-397 (Fig 5E). This protein modification is required for its full activation, which leads to the recruitment of focal adhesion proteins like vinculin and paxillin. Vinculin associates with FAK in elongated dot-like structures and couples focal adhesions with actin fibers. This structure could be observed in HUVEC treated with control medium. In contrast, Sema3C almost abolished the formation of such focal adhesion complexes (Fig 5F). The recruitment of paxillin, another focal adhesion protein, was also disturbed by Sema3C treatment (Supplementary Fig S5C). Collectively, the data indicate that Sema3C impaired the formation of focal adhesions that are necessary for cell adhesion and migration.

### Nrp-1 and Plexin-D1 act as receptors for Sema3C in endothelial cells

**Figure 6:**
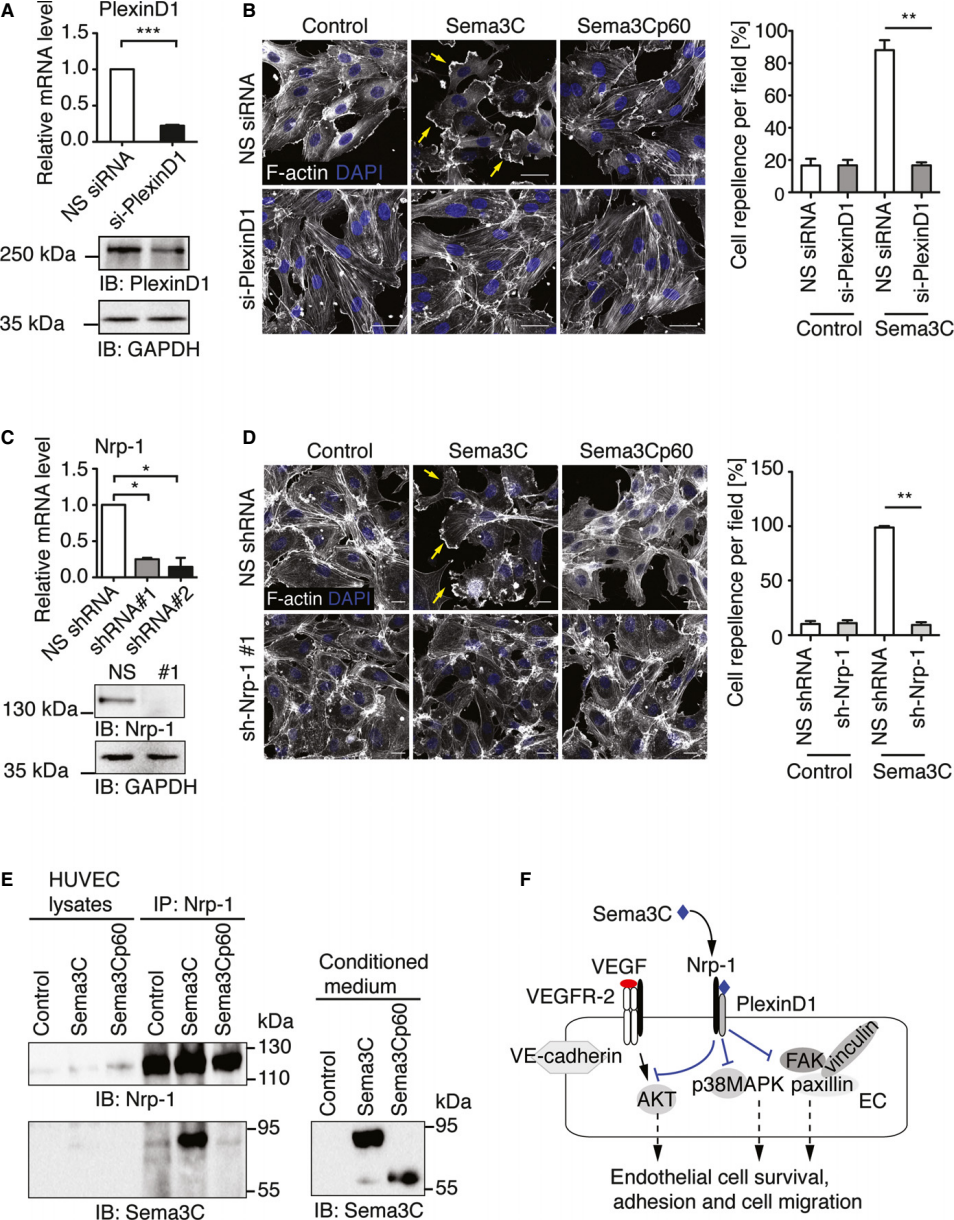
Sema3C, but not Sema3Cp60, acts through Neuropilin-1 and PlexinD1 to suppress endothelial cell adhesion HUVECs were transfected with siRNA against PlexinD1 or control NS (non-silence) siRNA. qPCR analyses and Western blotting were performed after 48 h (*n* = 3; paired Student's *t*-test, NS siRNA vs. PlexinD1 siRNA, ***P =* *0.0004). Cell lysates were subjected to SDS–PAGE and probed with anti-PlexinD1.
HUVECs were transfected with siRNA against PlexinD1 and plated on gelatin-coated coverslips. Confluent monolayers were treated with Sema3C-, Sema3Cp60- or control-conditioned medium for 30 min. Cells were fixed and stained with Alexa-Fluor 488-conjugated phalloidin to visualize F-actin. Arrows indicate Sema3C-induced cell membrane ruffling. Scale bar, 20 μm. Cell repellence was quantified as the number of cells with membrane ruffling normalized to the number of nuclei (*n* = 5; mean ± s.e.m., paired Student's *t*-test, **P =* *0.001).
Silencing of Nrp-1 by lentiviral particles expressing shRNA. After culturing 72 h in selection medium, RNA was harvested for qPCR analysis. NS shRNA vs. Nrp-1 shRNA: #1, *P =* *0.017 and #2, *P =* *0.02; *n* = 3; mean ± s.e.m. Cell lysates were subjected to SDS–PAGE and probed with anti-Nrp-1.
Depletion of Nrp-1 abolished the response to Sema3C. Sema3Cp60 had no effect on HUVEC adhesion. Arrows indicate membrane ruffling. Scale bar, 20 μm. Cell repellence was quantified as the number of cells with membrane ruffling normalized to the number of nuclei (*n* = 5; mean ± s.e.m.; paired Student's *t*-test, ***P =* *0.0003).
HUVECs were treated with Sema3C-conditioned medium for 10 min and lysed, and Nrp-1 was immunoprecipitated. Bound Sema3C was detected by Western blotting.
Schematic model of Sema3C signaling through Nrp-1 and PlexinD1 receptors in endothelial cells. Source data are available online for this figure.

Lastly, we pursued experiments aimed at identifying the receptor(s) through which Sema3C exerts its functions on endothelial cells. Previous studies showed that Sema3C induces growth cone collapse in sympathetic neurons through both Nrp-1 and Nrp-2 receptors (Chen *et al*, 1997) and that PlexinD1 promotes the binding of Sema3C to Nrp-1 in COS cells (Gitler *et al*, 2004). PlexinA2 appeared as a potential receptor for Sema3C during heart development (Brown *et al*, 2001). To identify the functional receptors for Sema3C on endothelial cells, HUVECs were transfected with siRNA against PlexinD1 or lentivirally transduced with shRNA targeting Nrp-1, Nrp-2, or PlexinA2. qPCR analysis and Western blot analysis confirmed a sufficient downregulation of gene expression (Fig 6A and Supplementary Fig S6A and B). The cells were then treated with Sema3C-conditioned medium or recombinant protein for 30 min and changes of cell morphology were analyzed. Sema3C caused the rapid disassembly of cytoskeletal actin fibers, while actin fibers became enriched at membrane ruffles (Fig 6B and D). Silencing of Nrp-2 and Plexin-A2 could not prevent cell detachment after Sema3C treatment (Supplementary Fig S6C), indicating that these receptors were not essential for Sema3C function in endothelial cells. However, silencing of PlexinD1 or Nrp-1 expression rendered endothelial cells resistant to Sema3C treatment (Fig 6B and D, Supplementary Fig S6D and E). Thus, our data clearly indicated that both Nrp-1 and PlexinD1 receptors are responsible for transducing Sema3C signals.

In contrast to Sema3C, the short Sema3Cp60 isoform had no effect on F-actin bundles (Fig 6B and D). Therefore, we tested Sema3C isoforms for their ability to directly interact with Nrp-1. To this end, HUVECs were first treated with Sema3C-, Sema3CΔ13-, and Sema3Cp60-conditioned media and Nrp-1 was immunoprecipitated. The bound Sema3C proteins were detected by immunoblotting. This revealed that Sema3C, but not the Sema3CΔ13 and Sema3Cp60 isoforms, interacted with Nrp-1 (Fig 6E and Supplementary Fig S6F). This was in line with the result that the short Sema3Cp60 isoform had no anti-angiogenic activity (Fig 1C and D), suggesting that cleavage within the PSI domain abrogated binding of Sema3C to the Nrp-1 receptor.

Taken together, these results demonstrate that Sema3C signals via Nrp-1 and PlexinD1 receptors on endothelial cells to impair the integrity of adherens junctions, the assembly of actin cytoskeleton, and focal adhesions. The inhibition of these critical cellular functions strongly impairs VEGF-promoted endothelial cell survival, cell adhesion, and cell migration during angiogenesis (Fig 6F).

### Sema3C suppresses pathological angiogenesis in an oxygen-induced retinopathy model

**Figure 7:**
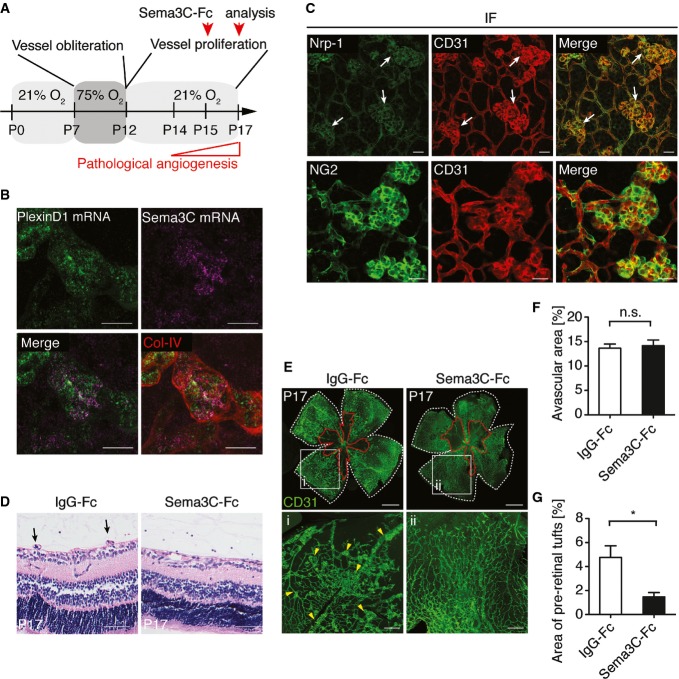
Sema3C inhibits pathological angiogenesis in an oxygen-induced retinopathy model in mice Scheme of the experiment. C57BL/6N mice pups at P7 with nursing mothers were subjected to hyperoxic conditions leading to vessel obliteration in the central zone of the retina. At P12, mice pups were returned to room air, which induces vessel regrowth but also pathological neovascularization, characterized by pre-retinal neovascular tufts. At P15, mice pups were injected intravitreally with 1 μg control IgG-Fc (left eye) and Sema3C-Fc (right eye). Retinae were analyzed 2 days later after intravitreal injection.
RNA scope analyses showed Sema3C and PlexinD1 expression in pre-retinal tufts (OIR model, P19). The retinae were subsequently stained against collagen IV. Sema3C mRNA was detected in peri-endothelial cells. Scale bar: 20 μm.
Nrp-1 expression in pre-retinal tufts (OIR model, P17). Abundant expression of Nrp1 in endothelial cells. Pericytes at vascular tufts were immunostained with anti-NG2. Scale bar, 20 μm.
H&E staining shows neovascular tufts (arrows). Representative of five retinae per group in two independent experiments. Scale bar, 50 μm.
Whole-mount CD31 immunostaining. The white line defines the whole retinal area, and red line outlines the vaso-obliterative zone. Inset image shows large numbers of peri-retinal tufts (i, arrow heads) in control IgG-Fc-treated retinae compared to Sema3C-treated retinae (ii). Scale bar, 500 μm (upper panel) and 100 μm (lower panel).
The avascular area is presented as percentage of the whole retinal area (*n* = 10 retinae in each group; n.s., non-significant using paired Student's *t*-test).
The formation of pre-retinal tufts was quantified in the superficial layer of the retinal vasculature and normalized to whole retinal area (*n* = 6 retinae in each group; *P = 0.033 using paired Student's *t*-test).

The human disease retinopathy of prematurity (ROP) is characterized by the rapid outgrowth of pathological blood vessels from the retina to the vitreous body. These pre-retinal tufts can lead to hemorrhage or retinal detachment, which often results in vision loss. The mouse OIR model reproduces both phases of ROP and can be used to test therapeutic substances or other treatment. Mice were exposed to hyperoxic conditions (75% oxygen) from postnatal day P7 to day P12. Oxygen toxicity and decreased VEGF levels cause vessel regression in the central retina resulting in the vaso-obliterative zone, which resembles phase 1 of ROP. At day P12, mice were returned to normal oxygen tension, and this relative hypoxic environment increased VEGF secretion leading to re-vascularization. However, this also drives the formation of pathological neovessel formation resulting in outgrowth of immature vessels into the vitreous body (pre-retinal tufts) (Stahl *et al*, 2010) (Fig 7A). As we showed that Nrp-1 and PlexinD1 are the functional receptors for Sema3C and since these two receptors are strongly upregulated in ROP (Oh *et al*, 2002; Fukushima *et al*, 2011), we also confirmed the increased expression of both receptors during physiological angiogenesis (Supplementary Fig S7A) and on pre-retinal tufts during OIR (Fig 7B and C, Supplementary Fig S7B). Interestingly, we found that expression of Sema3C mRNA was not detectable at early stages of retinal angiogenesis in physiological conditions (Supplementary Fig S7A) but increased during OIR. Here, Sema3C was most likely produced by peri-endothelial cells (Fig 7B). However, in retinal lysates, only slight amount of the p60 protein isoform was detectable (Supplementary Fig S7C), indicating rapid inactivation of the endogenous protein. Thus, we hypothesized that recombinant Sema3C might be a potent agent to interfere with pre-retinal tuft formation in the mouse OIR model.

To evaluate whether local Sema3C administration into the vitreous body provides therapeutic benefits, we injected a single dose of 1 μg recombinant murine Sema3C protein or control IgG-Fc intravitreally at day P15 and analyzed retinae at day P17 (Fig 7A), the time point when pathological vessel growth reaches its maximum.

H&E staining of retinal specimens showed the formation of hypoxia-driven pre-retinal vascular tufts that appeared in the control IgG-treated retinae. In contrast, mice treated with recombinant Sema3C developed almost no pre-retinal tufts (Fig 7D). The sizes of avascular vaso-obliterative zones were equal in both groups (Fig 7E and F). However, Sema3C treatment almost completely abolished pathological neoangiogenesis from the superficial vascular layer toward the vitreous body (Fig 7E and G).

Taken together, locally delivered Sema3C acted as a potent anti-angiogenic protein on immature vessels. If applied to the vitreous body, it prevented sprouting toward this direction and formation of pre-retinal tufts. This finding may open new avenues to treat diseases defined by the uncontrolled outgrowth of dysfunctional blood vessels.

## Discussion

Sprouting angiogenesis requires dynamic changes in endothelial cell shape, orientation, and adhesion. This is similar to the outgrowth of axons and it is not surprising that axonal guidance molecules coordinate both processes. This study showed for the first time that the secreted class-3 Semaphorin protein Sema3C inhibits sprouting angiogenesis by causing a substantial rearrangement of the actin cytoskeleton, breakdown of adherens junctions, and impairment of focal adhesion formation. Our data are in line with the functions of other semaphorins which act through Rho GTPases, FAK, and Src kinase family members in regulating cell spreading during neurite outgrowth, tumor progression, and vascular barrier integrity (Barberis *et al*, 2005; Franco & Tamagnone, 2008; Shimizu *et al*, 2008; Le Guelte *et al*, 2012). Here, we identified Sema3C functions that impeded VEGF-mediated endothelial cell survival and cell migration and finally caused cell detachment and apoptosis. This was seen in cellular models as well as *in vivo*. However, the few vessels that were formed by transplanted endothelial cells in the presence of Sema3C were covered by mural cells as in control animals. This is similar to the function of Sema3A, which acts anti-angiogenic but also increases pericyte coverage in a pancreatic tumor model (Maione *et al*, 2012). Notably, Sema3C did not influence cell survival of pericytes. It was reported that PlexinD1 is only expressed in the growing vasculature (within tumors) but not in non-malignant mature blood vessels (Roodink *et al*, 2009). This is in line with our expression data of Nrp-1 and PlexinD1 in the retina; hence, we suggest that Sema3C is only capable of driving such vessels into apoptosis that are undergoing angiogenesis and remodeling. This was further supported by the finding that quiescent HUVEC on Xellulin hydrogels were protected from Sema3C-induced cell repellence.

Exaggerated secretion of VEGF, Epo, Angiopoietin-2, and metabolic factors like succinate is responsible for the excessive growth of retinal blood vessels toward vitreous and lens during retinopathy of prematurity (ROP) (Sapieha *et al*, 2010). This disease is difficult to treat since on the one hand revascularization of the vaso-obliterative zone is desirable, whereas sprouting angiogenesis toward the vitreous body may lead to retinal detachment, hemorrhage, and blindness. The local injection of a single dose of recombinant Sema3C protein into the vitreous body in a mouse model of oxygen-induced retinopathy did not change the size of the vaso-obliterative zone within the retina. However, Sema3C effectively prevented the formation of pathological pre-retinal tufts that are responsible for major retinal damage. This inhibition was stronger than that observed after the local administration of Nrp-1 blocking antibodies (Oh *et al*, 2002). Importantly, Sema3C did not cause any changes in vascular morphology of mature retinal vessels, a crucial safety factor for any therapeutic agent targeting pathological angiogenesis. This may be due to the fact that our data and also previous study showed that the expression of both Sema3C receptors Nrp-1 and PlexinD1 is highly expressed in tip cells during physiological angiogenesis (Oh *et al*, 2002; Kim *et al*, 2011) and during pathological angiogenesis (Fukushima *et al*, 2011). Therefore, our study suggests that Sema3C/Nrp-1/PlexinD1 signaling represents an attractive target for anti-angiogenic therapy.

Semaphorin signal transduction requires the association of Plexin and Neuropilin receptors. The presence of different receptor combinations mediates differential functions of the ligands. As such, Sema3C repels cortical neurons through Plexin/Nrp-1 but attracts neurons expressing Plexin/Nrp-2 receptor complexes (Ruediger *et al*, 2013). Though it was reported that Sema3C could act via both Neuropilins in sympathetic neurons (Chen *et al*, 1998), our data revealed that Sema3C selectively acted through PlexinD1 and Nrp-1 on endothelial cells. This is compatible with mouse knockout studies. Sema3C^−/−^ mice die perinatally with cardiac defects (Feiner *et al*, 2001), which is similar to the phenotype observed in endothelial-specific Nrp-1-deficient or PlexinD1-deficient mice (Gu *et al*, 2003; Gitler *et al*, 2004). However, PlexinD1^−/−^ or Nrp-1^−/−^ mouse embryos show additional vascular patterning defects and several other abnormalities (Kawasaki *et al*, 1999; Gitler *et al*, 2004). This may be due to the fact that both receptors also mediate the signals of other ligands including VEGF (Soker *et al*, 1998).

This study also demonstrated that proteolytic processing controls binding of Sema3C to its receptor. We found that the short Sema3Cp60 isoform had no anti-angiogenic activity, which is similar to the effect of cleaved Sema3B (Varshavsky *et al*, 2008). Mechanistically, we found that the Sema3Cp60 isoform did not bind to the Nrp-1 receptor indicating that the other domains of Sema3C are of utmost functional importance. The Sema3CΔ13 isoform, which may result from metalloproteinase cleavage, showed, however, strong anti-angiogenic activity. This is similar to a study that describes strong activity of the Sema3CΔ13 isoform in a model of cancer cell migration (Esselens *et al*, 2010). Furin cleavage of semaphorins can lead to the exposure arginine/proline residues at the C-terminus, which are critical for ligand–receptor engagement (c-end rule) (Parker *et al*, 2013). The impaired binding of Sema3CΔ13, which lacks this C-terminal motif, to Nrp-1 in a co-immunoprecipitation experiment supports the importance of the C-end rule. However, it was also shown that the Ig domain of Sema3C could bind both Nrp-1 and Nrp-2 (Chen *et al*, 1998). Therefore, it may well be that Sema3CΔ13 executes its activity through binding to Nrp-1 with lower affinity, which could not be demonstrated in this experimental setup.

In summary, this study demonstrates for the first time that Sema3C can act as a potent anti-angiogenic agent that suppresses sprouting angiogenesis through dual inhibition of endothelial junction integrity and focal adhesion with the extracellular matrix. Moreover, administration of Sema3C effectively inhibits the formation of pathological vessel growth, such as pre-retinal neovascular tufts in the ROP model. Therefore, our study shows for the first time that Sema3C is a promising agent to selectively interfere with pathological angiogenesis in the retina.

## Materials and Methods

### Plasmids, shRNA, and RNAi

Sema3C cDNA (NCBI DQ890847) vector from Genomic and Proteomics Core Facility of German Cancer Research Center was shuttled into pAD/CMV/V5 and pLenti6.2/V5-DEST (Invitrogen) by Gateway cloning for virus production. Sema3CΔ13 and Sema3Cp60 isoforms were generated using the QuickChange Lightening Site-Directed Mutagenesis Kit (Agilent Technologies) to introduce stop codons. PCR primers for the Sema3CΔ13 isoform: (5′-CAAGTTAAAGGCCCTCATCAATAGTTAGTAAAGTAGAAACAGGAGGAATCAGTT-3′), (5′-AACTGATTCCTCCTGTTTCTACTTTACTAACTATTGATGAGGGCCTTTAACTTG-3′); and for Sema3Cp60: (5′-GAAACGGAGGAGCCGAAGATAATAGGTGAGACATGGAAACCCAC-3′) and (5′-GTGGGTTTCCATGTCTCACCTATTATCTTCGGCTCCTCCGTTTC-3′). The translated protein sequences are shown in Fig 1A. Control siRNA (AM4636) and siRNA against human plexinD1 (AM16708, clone ID: 108678) were from LifeTechnologies. Transfection of HUVEC (1.2 × 10^5^ cells) with siRNA duplexes (final concentration, 200 pmol) was performed with Oligofectamine according to the transfection protocol (Invitrogen). shRNA against Nrp-1 (clone ID: 16466, 333723) and Nrp-2 (clone ID: 346687, 176015) were from Thermo Scientific. shRNA against PlexinA2 (clone ID: 389145, 389146) was from Dharmacon.

### RNA isolation and qPCR analysis

RNA was purified with the RNeasy Kit (Promega) and transcribed into cDNA (High Capacity cDNA Reverse Transcription Kit; Life Technologies). Real-time PCR was performed using the STEPOnePlus real-Time PCR system (Applied Biosystems). GAPDH and OAZ1 served as housekeeping genes for normalization. Primer sequences are listed in Table 1.

**Table 1:** Primer sequences for real-time PCR analysis

Gene		Sequence
APLN	forward 5′-3′	CTCTGGCTCTCCTTGACCG
	reverse 5′-3′	GAATTTCCTCCGACCTCCCT
PDGFB	forward 5′-3′	CTTTAAGAAGGCCACGGTGA
	reverse 5′-3′	CTAGGCTCCAAGGGTCTCCT
ESM1	forward 5′-3′	CTTGCTACCGCACAGTCTCA
	reverse 5′-3′	ACTGGCAGTTGCAGGTCTCT
ANGPT2	forward 5′-3′	GGGAAGGGAATGAGGCTTAC
	reverse 5′-3′	AAGTTGGAAGGACCACATGC
DLL4	forward 5′-3′	GCCTGGACAAGTCCAACTGT
	reverse 5′-3′	CGCTGATATCCGACACTCTG
HEY1	forward 5′-3′	GAGAAGGCTGGTACCCAGTG
	reverse 5′-3′	CGAAATCCCAAACTCCGATA
HEY2	forward 5′-3′	CTTGTGCCAACTGCTTTTGA
	reverse 5′-3′	GCACTCTCGGAATCCTATGC
EFNB2	forward 5′-3′	CTGCTGGATCAACCAGGAAT
	reverse 5′-3′	GATGTTGTTCCCCGAATGTC
GAPDH	forward 5′-3′	ATGTTCGTCATGGGTGTGAA
	reverse 5′-3′	GTCTTCTGGGTGGCAGTGAT
OAZ1	forward 5′-3′	GAGCCGACCATGTCTTCATT
	reverse 5′-3′	CTCCTCCTCTCCCGAAGACT
NRP1	forward 5′-3′	CGAAATCGGAAAAGGAAACC
	reverse 5′-3′	ATCCAGGTCTGCTGGTTTTG
NRP2	forward 5′-3′	GAAGAGGAGGCCACAGAGTG
	reverse 5′-3′	CCGCAAGAAATTCCTGTCAT
PLXND1	forward 5′-3′	AACATCTCCAGCCAGAGCAG
	reverse 5′-3′	CCAGGAAGACCGCTGTGTAG
PLXNA2	forward 5′-3′	GCCATCAACTTGCAGATCAA
	reverse 5′-3′	GTAGGAGGCCACAGAGGTCA

### Cell culture

HBMEC (Cell Systems) were maintained in BMEC growth media (PELO Biotech). HUASMC were cultured in high-glucose DMEM containing 15% FCS. HUVECs were grown and maintained until passage 5 in Endopan3 Growth Medium containing supplements (Pan-Biotech). Human brain pericytes (ScienCell Research Lab) were cultured in pericyte medium containing supplements (ScienCell Research Lab) and used between passages 3 and 7. Sema3C-conditioned medium was prepared 24 h after adenoviral infection of pericytes. The medium was replaced with Endopan3 basal medium (0.5% FCS) for additional 48 h. Conditioned medium was centrifuged at 201 *g* for 5 min and directly applied to HUVEC.

### Antibodies and reagents

Recombinant human VEGF_165_ (Ala27-Arg191, no. 293-VE), human Sema3C (Gly21-Ser738, no. 5570-S3), and mouse Sema3C (Gln24-Ser741, no. 1728-S3) were from R&D Systems. Recombinant human IgG-Fc (009-000-008) was purchased from Jackson ImmunoResearch. Antibodies: rat anti-Sema3C (clone 238835) and goat anti-Nrp-1 (AF566) are from R&D Systems; rabbit anti-pY397FAK, rabbit anti-pT180/pY182-p38MAPK (Invitrogen); rabbit anti-FAK (C-20), and goat anti-Nrp-1 (C-19) are from Santa Cruz; mouse anti-p38MAPK (L53F8), rabbit anti-AKT, rabbit anti-pS473AKT, and rabbit anti-cleaved Caspase-3 (Asp175) are from Cell Signaling; pTyr118 paxillin (NEB), rat anti-CD34 (NCL-END; Novocastra); rat anti-CD31 (clone Mec13.3) and rat anti-VE-cadherin are from BD Biosciences; mouse anti-vinculin (V9131) and anti-actin smooth muscle-Cy3 are from Sigma; rabbit anti-GFAP (DAKO), rabbit anti-NG2 (Millipore), rabbit anti-collagen IV (Cosmo Bio).

### Cell proliferation, migration, and apoptosis

HUVECs were labeled by BrdU labeling solution (Roche) and treated with conditioned medium for 24 h. The level of incorporated BrdU was measured at OD_370_ with an ELISA reader (TECAN). To measure cell migration, HUVECs (20,000 cells) were seeded into the lower transwell with the plastic insert (ibidi) and cultured overnight. Pericytes were infected with adenoviral vector expressing Sema3C or GFP as a control and grown on transwells for 24 h. A gap within the HUVEC monolayer was generated by removing the inserts, and the growth medium was replaced with Endopan3 basal medium (0.5% FCS). At the same time, pericytes were cocultured with HUVEC. The gap in the HUVEC monolayer was imaged with an Olympus IX 50 microscope at 4× magnification at 0 and 24 h. Bright-field images were taken and analyzed with Cell^P software. Apoptosis was measured with the Caspase-Glo®3/7 Substrate solution (Sigma) after 3 h of incubation of conditioned medium.

### Immunofluorescent staining

HUVECs (6 × 10^4^ cells) were cultured on 0.2% gelatin-coated coverslips for 48 h. Cells were starved in basal medium containing 0.5% FCS for 16 h before treatment with conditioned medium. Cells were fixed in 4% PFA, permeabilized with 0.3% Triton X-100 in PBS, and incubated in 3% BSA in PBS for 1 h. pY397FAK, vinculin, or pY118 paxillin antibodies (1:100 in blocking solution) were incubated overnight at 4°C. Coverslips were washed in PBS with 0.2% Tween-20, incubated with Alexa-488 goat anti-rabbit or Alexa-647 goat anti-mouse (Invitrogen) for 30 min. After a second washing step, samples were incubated with Alexa Fluor 546-conjugated phalloidin (Invitrogen) for 30 min. Images were acquired using a Zeiss LSM700 confocal microscope.

### Time-lapse imaging

HUVECs were transduced with viral vectors expressing GFP-VE-cadherin or RFP-LifeAct overnight, and 3 × 10^4^ cells were seeded into ibidi wells (ibidi). The next day, cells were observed using a wide-field microscope (Zeiss Cell Observer) equipped with an incubation chamber (5% CO_2_ at 37°C). The serial images were analyzed using ImageJ (Fiji) software.

### Kinase activation and immunoprecipitation

HUVECs (2.4 × 10^5^ cells) were cultivated for 48 h to reach full confluence and then starved in basal medium (0.5% FCS) for additional 16 h. Cells were treated with conditioned medium with or without 50 ng/ml VEGF_164_ (R&D Systems). Cells were lysed with 0.5% NP-40 containing protease and phosphatase inhibitors (Roche) on ice. Denatured protein lysates were then applied for SDS–PAGE. For immunoprecipitation of endogenous Nrp-1, serum-starved HUVECs were treated with conditioned medium for 10 min, washed with ice-cold PBS, and lysed with 0.5% NP-40 lysis buffer containing protease and phosphatase inhibitors on ice for 1 h. After centrifugation, the supernatant was pre-incubated with Protein-G PLUS-agarose (Santa Cruz) for 30 min. The supernatant was then incubated with 2 μg Nrp-1 antibody and Protein-G PLUS-agarose at 4°C overnight. Precipitated agarose beads were washed with ice-cold PBS and denatured in Laemmli sample buffer at 95°C for 5 min. Samples were then subjected to Western blotting.

### Tube formation and spheroid-based sprouting angiogenesis

Growth factor-reduced Matrigel (BD Biosciences) was added to 48-well plates to polymerize at 37°C for 30 min. HUVECs (2.5 × 10^4^ cells) were suspended in 260 μl conditioned medium. Cells were mixed with VEGF (25 ng/ml), and 250 μl solution was plated on top of Matrigel. The assay was terminated after 18 h by adding 4% PFA.

HUVEC spheroids were embedded in collagen-based gels as previously described (Brutsch *et al*, 2010) and stimulated with 100 μl conditioned medium with or without VEGF (25 ng/ml) for 24 h. Alternatively, human IgG-Fc or Sema3C-Fc (500 ng/ml) was added. The vascular structures were examined with a wide-field microscope (Olympus IX50) at 4× magnification and analyzed with Cell^P software.

### Endothelial cell xenotransplantation

Human brain pericytes were stably transduced with lentivirus expressing Sema3C or GFP at MOI = 10. After selection with 10 μg/ml blasticidin, an equal number of pericytes and HUVECs were mixed to generate spheroids. These were mixed with 300 μl growth factor-enriched Matrigel (BD Biosciences) containing fibrinogen, thrombin, VEGF_164_, and FGF2 (R&D systems) and injected s.c. into 8-week-old female CB17/SCID mice (Charles River) as previously described (Alajati *et al*, 2008). Before plugs were dissected after 28 days, 0.15 mg FITC-lectin was i.v. injected. PFA-fixed plugs were paraffin-embedded. Serial sections (10 μm) were stained with anti-human CD34 (1:100) and anti-SMA (1:100) (Laib *et al*, 2009). Images were acquired with a Zeiss LSM700 confocal microscope at 10× magnification. The vessel area was quantified as the CD34-positive area divided to the total plug area. Vessel coverage was quantified as αSMA-Cy3-positive pixels which are in direct proximity to CD34-positive endothelial cells divided through the vessel area. The number of co-localized cleaved caspase-3 and CD34 stained cells was counted manually and normalized to the number of CD34-positive cells.

### Whole-mount *in situ* hybridization (ISH) and RNAscope

For PlexinD1 ISH, the procedure was described previously (Fukushima *et al*, 2011). Briefly, enucleated retinae were fixed in 4% PFA at RT for 15 minutes and post-fixed in 4% PFA and 0.2% glutaraldehyde. Samples were hybridized with digoxigenin-labeled cRNA probes at 65°C overnight. The templates for cRNA probe: mouse PlexinD1, nt 5,184–6,055 of GenBank AK129175. The retinae were washed and incubated with alkaline phosphatase-conjugated sheep anti-digoxigenin antibody (Roche) at 4°C overnight. Bound antibodies were visualized using nitro blue tetrazolium and 5-bromo-4-chloro-3′-indolyphosphate (NBT/BCIP, Roche) as substrate. After ISH, the retinae were immunostained with polyclonal rabbit anti-collagen IV antibody, followed by labeling with Cy3-conjugated donkey anti-rabbit secondary antibody (Jackson ImmunoResearch). Images were taken with an Axioplan 2 microscope (Zeiss) equipped with associated software (AxioVision, version 3.1; Zeiss). RNAscope ISH (Advanced Cell Diagnostics) was modified from the protocol described previously (Gross-Thebing *et al*, 2014). Retinae were fixed in 4% PFA for 2 h at RT. Samples were hybridized with RNA target probes (PlexinD1, nt 1,555–2,414 of NM_026376.3, Channel 1; Sema3C, nt 906–1,841 of NM_013657.5, Channel 3) overnight at 40°C. The retinae were further immunostained with anti-collagen IV antibody. Images were taken with a confocal microscope (Zeiss LSM 700).

### Retinal angiogenesis and oxygen-induced retinopathy

Intravitreal injection of 1 μl recombinant mouse Sema3C fused to IgG-Fc (1 μg/μl) into the right eye and 1 μl of control human IgG-Fc into the left eye was performed in C57BL/6N mice pups at postnatal day P3 or in 6-week-old male mice. Two days later, the retinae were dissected and fixed in ice-cold methanol overnight. Samples were permeabilized in permeabilizing buffer (0.5% Triton X-100 and 10% donkey serum in PBS) for 1 h and were blocked in blocking buffer (0.2% Tween-20, 10% donkey serum) for 1 h. Samples were then stained with anti-CD31 antibodies (1:100) in PBS overnight at 4°C. After washing with PBS (0.2% Tween-20), the retinae were incubated with Alexa Fluor 488 donkey anti-rat IgG (H + L) antibody for 1 h. After washing steps, samples were mounted using fluorescence mounting medium (DAKO). For evaluating cell apoptosis, retinae were collected 8 h after injection of recombinant proteins and fixed in ice-cold methanol. Samples were incubated with anti-cleaved caspase-3 (1:50) and anti-CD31 (1:100). For investigating endothelial cell junctions, retinae were fixed in methanol 6 h after injection. Z-stack images were acquired using a confocal microscope (Zeiss LSM 700).

Oxygen-induced retinopathy was induced by placing C57BL/6N mice pups with the nursing mother at day P7 in an Oxycycler (BioSpherix) with 75% oxygen for 5 days. Mice returned to room air at P12 and received intravitreal injections of 1 μg recombinant Sema3C (right eye) or IgG-Fc (left eye) at P15. The retinae were collected and analyzed 2 days later. All immunofluorescence images were taken using a confocal microscope (Zeiss LSM 700) and analyzed with Fiji software. For H&E staining, eyes were fixed in 4% PFA overnight, dehydrated, and embedded in paraffin. Staining was performed using 6-μm-thick slides. Images were taken with a wide-field microscope (Zeiss Cell Observer) at 20× magnification.

All animal experiments were performed according to the guidelines of the local institution (German Cancer Research Center Heidelberg) and the local government (Regierungspräsidium Karlsruhe, Germany).

## The paper explained

### Problem

Excessive blood vessel formation is involved in several diseases, most notably malignant tumors, chronic inflammation, and eye diseases. Preterm babies who receive ventilation are at risk to develop retinopathy of prematurity (ROP). Increased oxygen levels first cause degeneration of the still developing immature blood vessels in the retina. This may be followed by outgrowth of new vessels toward the vitreous body. These abnormal pre-retinal neovascular tufts damage the retina and this can even lead to blindness. There is evidence that some neuronal guidance proteins of the Semaphorin gene family regulate angiogenesis. However, the functional roles of Sema3C are not fully understood, and it is unclear whether recombinant Sema3C could be applied to interfere with pathological angiogenesis.

### Results

We have demonstrated that Sema3C is an attractive agent to specifically inhibit the formation of pre-retinal neovascular tufts in an animal model of retinopathy of prematurity. We found the local administration of this protein selectively inhibits the formation of immature blood vessels while not affecting the already established ones. This study also identified the receptors through which Sema3C acts and how this leads to breakdown of endothelial cell contacts, cell detachment, and cell death.

### Impact

This study shows Sema3C is a potential therapeutic agent that selectively targets immature vessels, which express high levels of Sema3C receptors. This may open new avenues to interfere with eye diseases that involve the excessive growth of new blood vessels.

## Supporting Information

Supplementary Movie S4

Review Process File
