# Supplementary material for: Semaphorin-3C signals through Neuropilin-1 and PlexinD1 receptors to inhibit pathological angiogenesis
Source: EMBO Mol Med. 2015 Jul 20;7(10):1267–84. doi: 10.15252/emmm.201404922 (PMC4604683; doi:10.15252/emmm.201404922)

Semaphorin-3C signals through Neuropilin-1 and PlexinD1 receptors to inhibit pathological angiogenesis

Wan-Jen Yang[1,2,6], Junhao Hu[3,6], Akiyoshi Uemura[4], Fabian Tetzlaff[1], Hellmut G. Augustin[2,3] and Andreas Fischer[1,2,5,*]

Supplemental Data

Figures S1 – S7

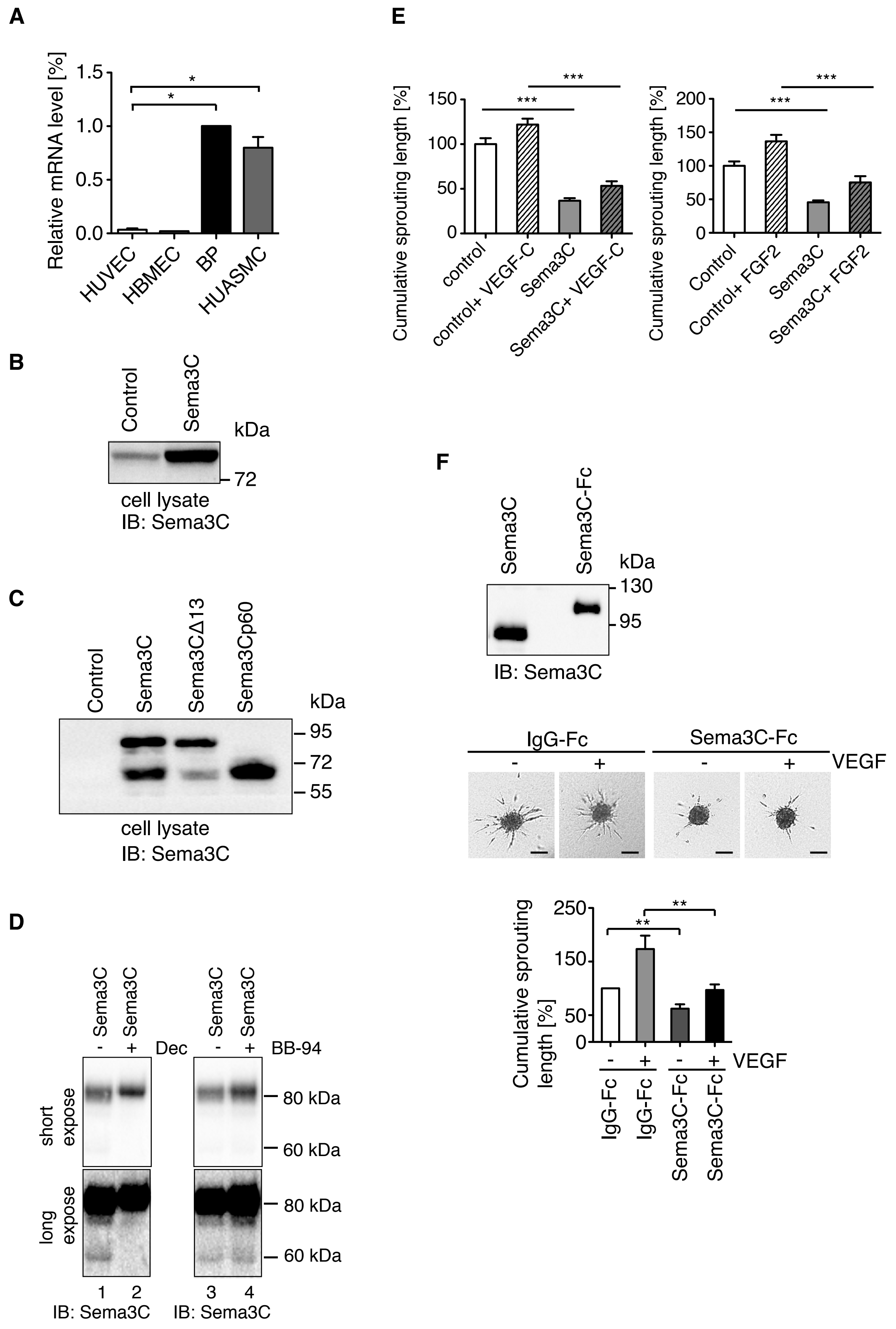
A
Relative mRNA level [%]
1.5
1.0
0.5
0.0
*
*
HUVEC
HBMEC
BP
HUASMC
B
Control
Sema3C
kDa
72
cell lysate
IB: Sema3C
C
Control
Sema3C
Sema3CΔ13
Sema3Cp60
kDa
95
72
55
cell lysate
IB: Sema3C
D
Sema3C
Sema3C
-
+
Dec
Sema3C
Sema3C
-
+
BB-94
short expose
long expose
80 kDa
60 kDa
80 kDa
60 kDa
1
2
3
4
IB: Sema3C
IB: Sema3C
E
Cumulative sprouting length [%]
150
100
50
0
***
***
control
control+ VEGF-C
Sema3C
Sema3C+ VEGF-C
Cumulative sprouting length [%]
200
150
100
50
0
***
***
Control
Control+ FGF2
Sema3C
Sema3C+ FGF2
F
Sema3C
Sema3C-Fc
kDa
130
95
IB: Sema3C
IgG-Fc
Sema3C-Fc
-
+
-
+
VEGF
Cumulative sprouting length [%]
250
150
50
0
**
**
-
+
-
+
VEGF
IgG-Fc
IgG-Fc
Sema3C-Fc
Sema3C-Fc

Supplementary Figure 2 **Yang et al.**

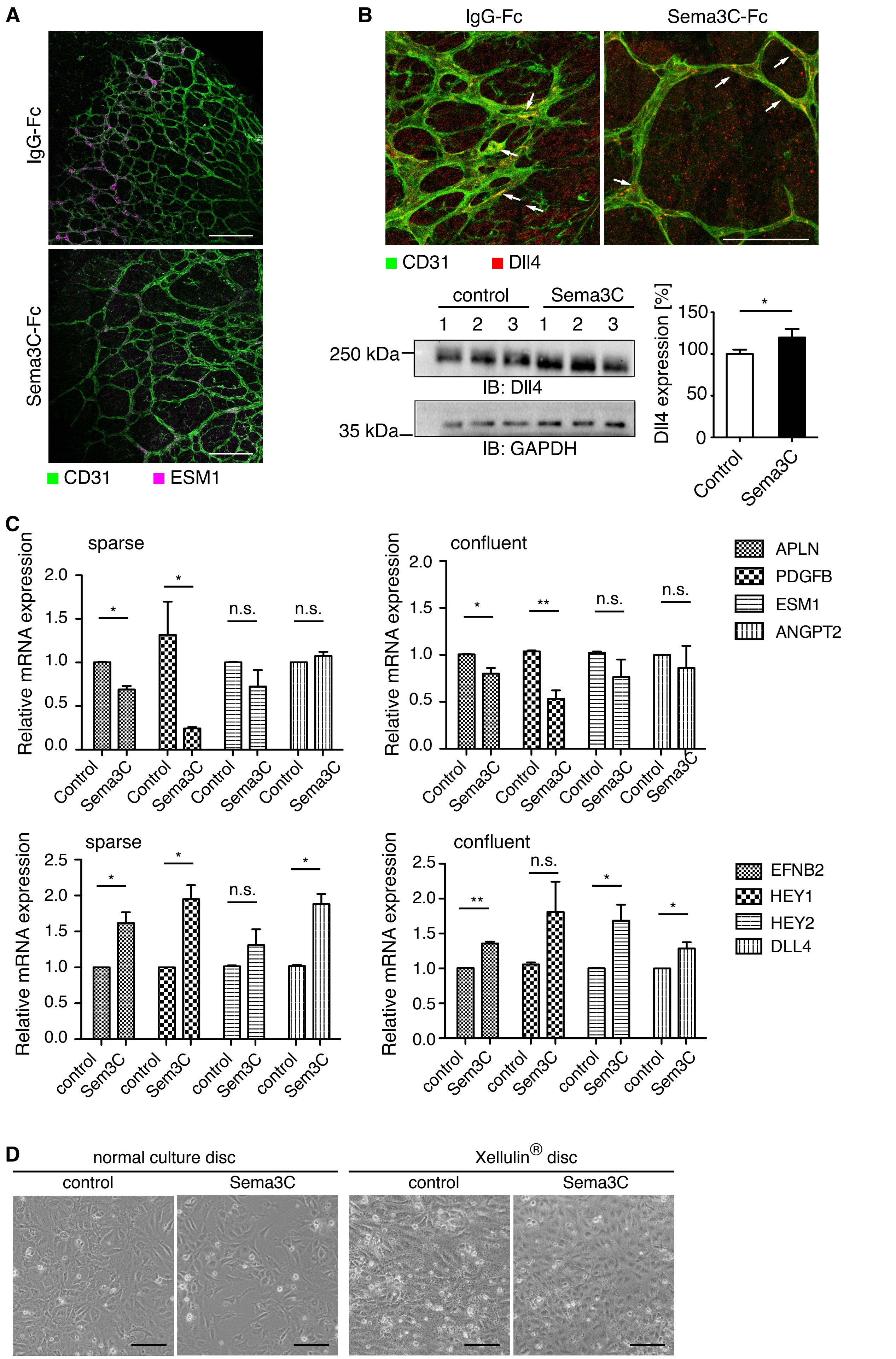

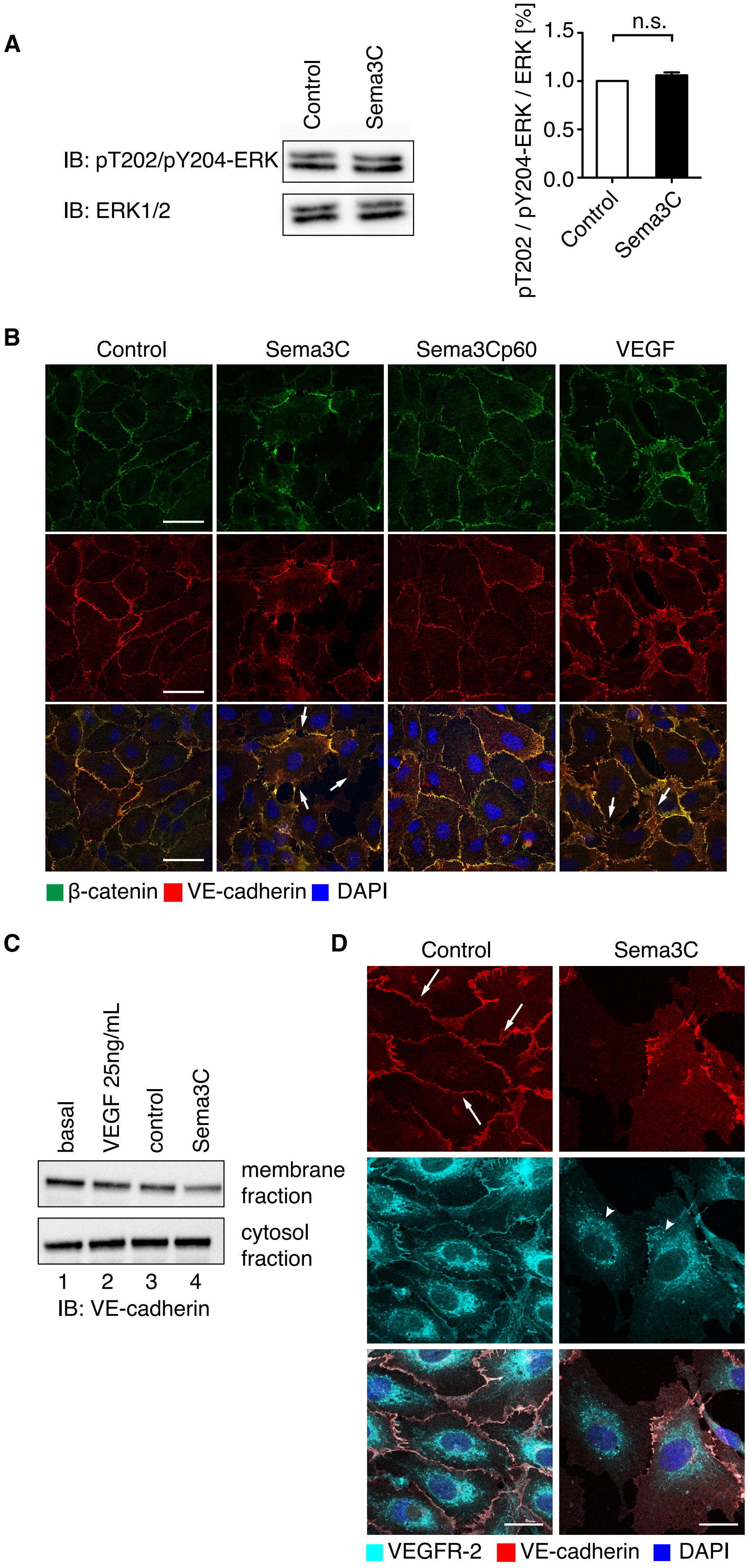
A
Control
Sema3C
IB: pT202/pY204-ERK
IB: ERK1/2
pT202 / pY204-ERK / ERK [%]
1.5
1.0
0.5
0.0
n.s.
Control
Sema3C
B
Control
Sema3C
Sema3Cp60
VEGF
β-catenin VE-cadherin DAPI
C
basal
VEGF 25ng/mL
control
Sema3C
membrane fraction
cytosol fraction
1 2 3 4
IB: VE-cadherin
D
Control
Sema3C
VEGFR-2 VE-cadherin DAPI

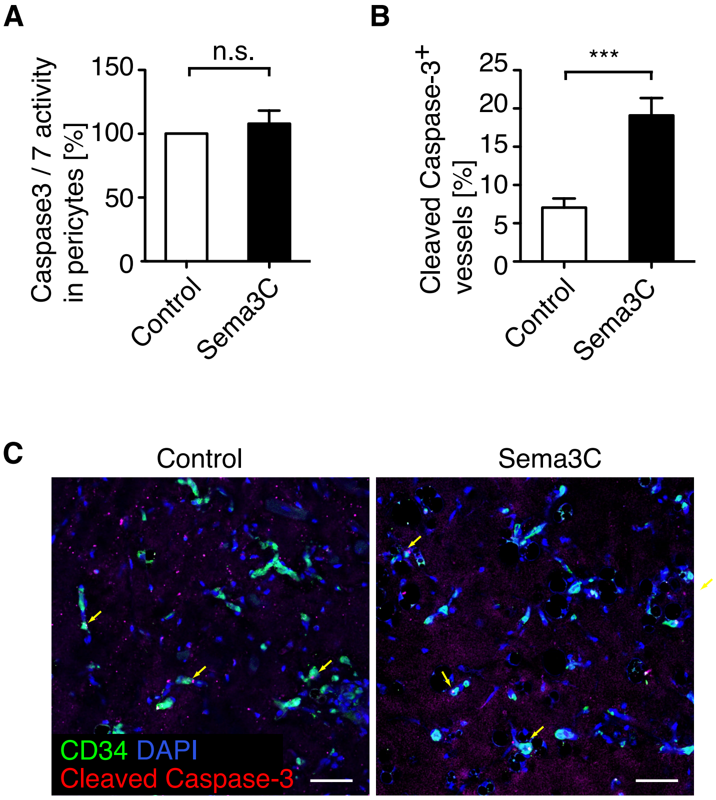
A
Caspase3 / 7 activity in pericytes [%]
150
100
50
0
n.s.
Control
Sema3C
B
Cleaved Caspase-3+ vessels [%]
25
20
15
10
5
0
***
Control
Sema3C
C
Control
Sema3C
CD34 DAPI
Cleaved Caspase-3

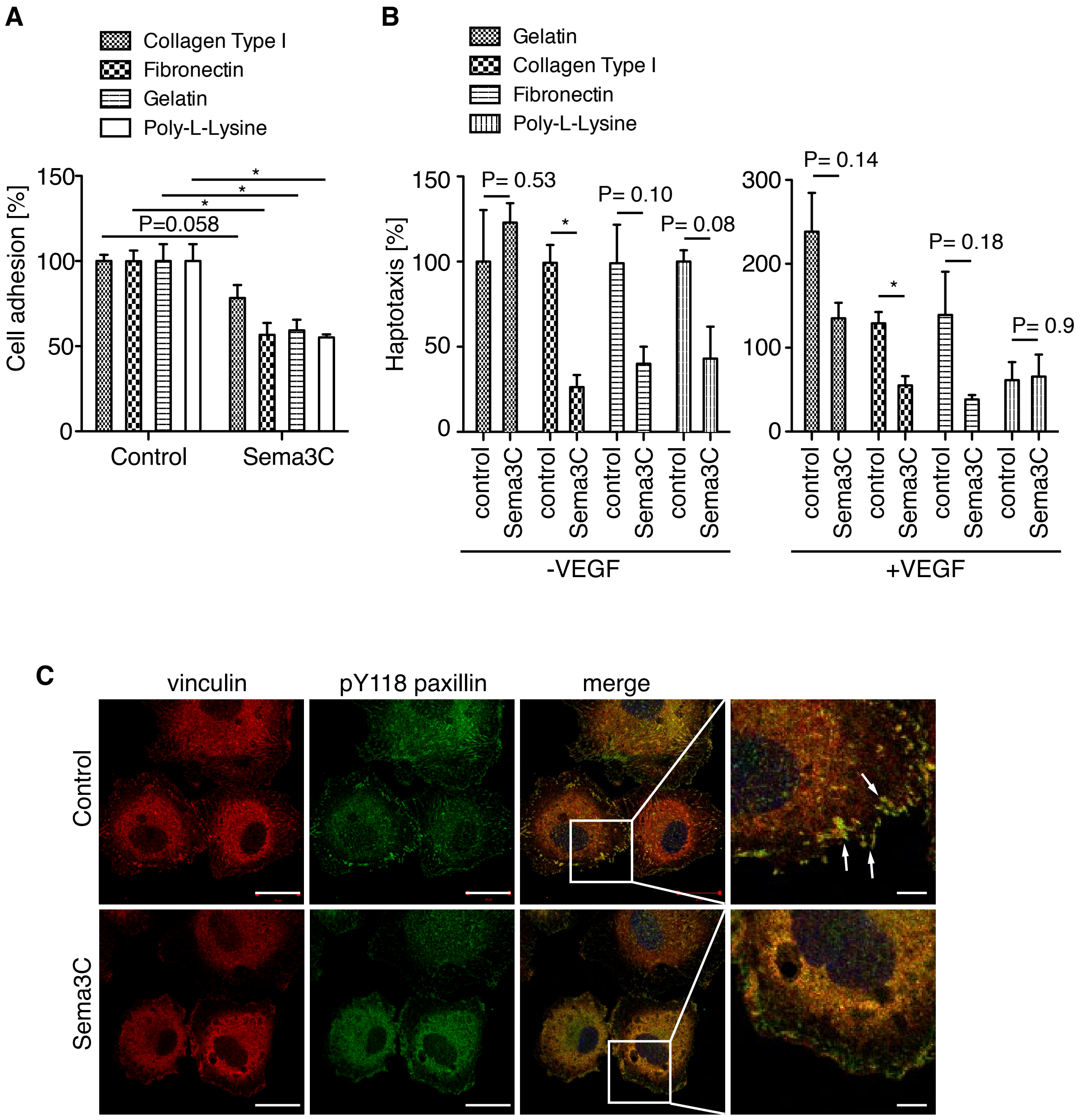
A
Collagen Type I
Fibronectin
Gelatin
Poly-L-Lysine
Cell adhesion [%]
150
100
50
0
P=0.058
*
*
*
Control
Sema3C
B
Gelatin
Collagen Type I
Fibronectin
Poly-L-Lysine
Haptotaxis [%]
150
100
50
0
P= 0.53
*
P= 0.10
P= 0.08
300
200
100
0
P= 0.14
*
P= 0.18
P= 0.9
control
Sema3C
control
Sema3C
control
Sema3C
control
Sema3C
-VEGF
control
Sema3C
control
Sema3C
control
Sema3C
control
Sema3C
+VEGF
C
vinculin
pY118 paxillin
merge
Control
Sema3C

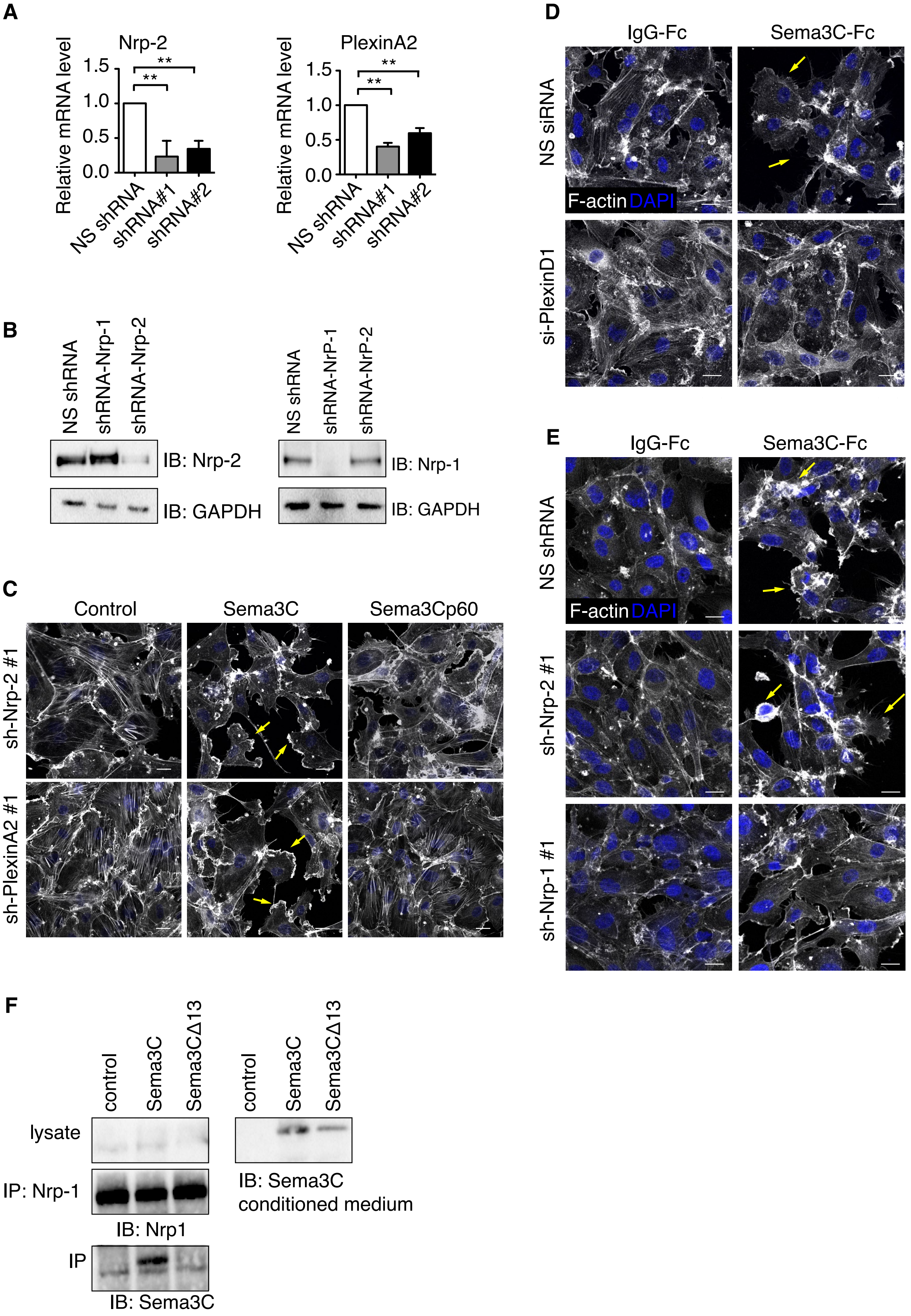
A
Nrp-2
Relative mRNA level
1.5
1.0
0.5
0.0
**
**
NS shRNA
shRNA#1
shRNA#2
PlexinA2
B
NS shRNA
shRNA-Nrp-1
shRNA-Nrp-2
IB: Nrp-2
IB: GAPDH
shRNA-NrP-1
shRNA-NrP-2
IB: Nrp-1
C
Control
Sema3C
Sema3Cp60
sh-Nrp-2 #1
sh-PlexinA2 #1
D
IgG-Fc
Sema3C-Fc
NS siRNA
si-PlexinD1
F-actin DAPI
E
NS shRNA
sh-Nrp-1 #1
F
control
Sema3C
Sema3CΔ13
lysate
IP: Nrp-1
IB: Nrp1
IP
IB: Sema3C
IB: Sema3C
conditioned medium

Supplementary Figure 7 Yang et al.

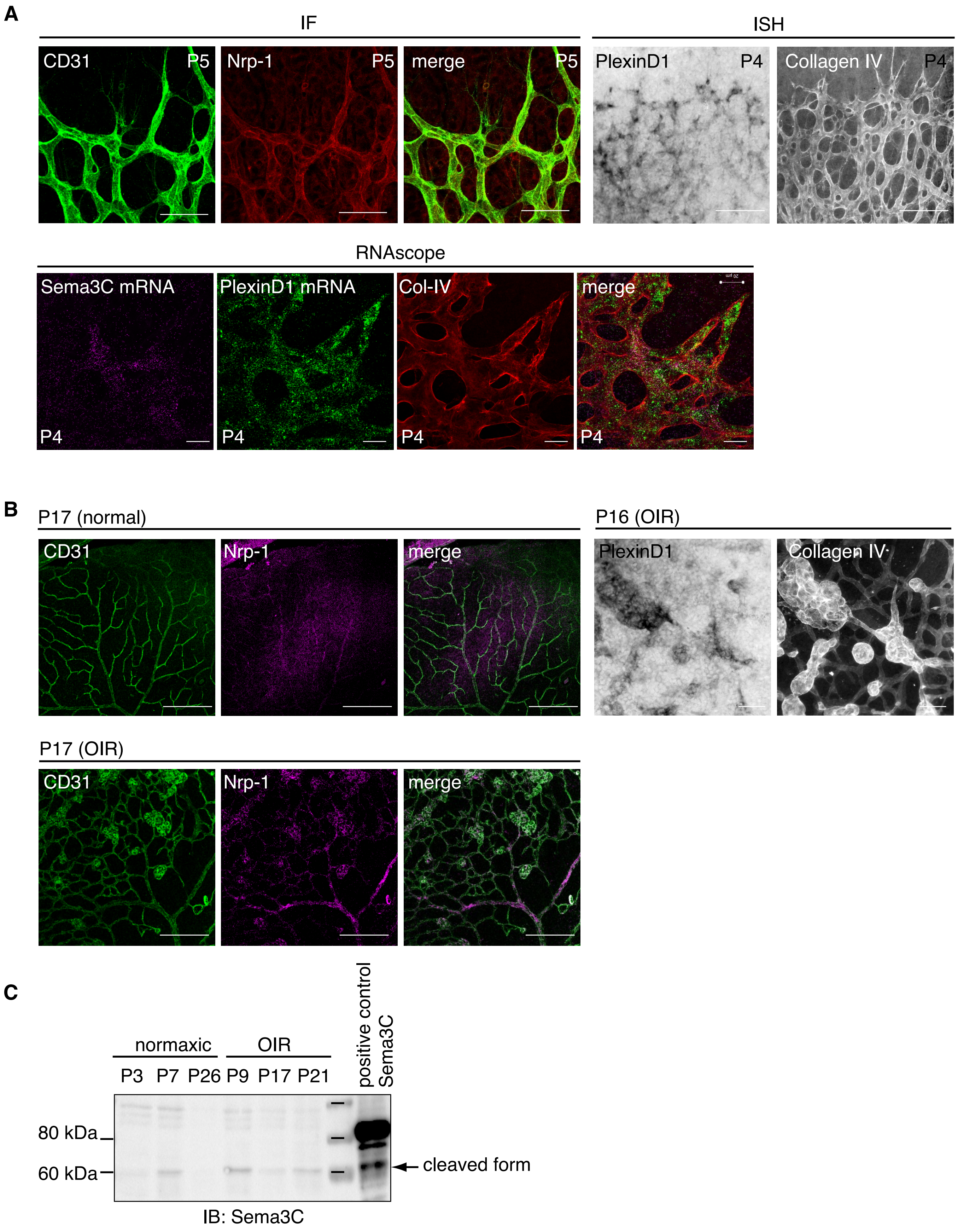

Supplement: Supplementary file 1 [file emmm0007-1267-sd1.pdf]
