# Supplementary material for: Semaphorin-3C signals through Neuropilin-1 and PlexinD1 receptors to inhibit pathological angiogenesis
Source: EMBO Mol Med. 2015 Jul 20;7(10):1267–84. doi: 10.15252/emmm.201404922 (PMC4604683; doi:10.15252/emmm.201404922)
Supplement: Supplementary file 2 [file emmm0007-1267-sd2.docx]

**Supplementary Figure Legends**

**Supplementary Fig. 1**

(**A**) mRNA expression of Sema3C was evaluated by qPCR in human umbilical venous endothelial cells (HUVEC), human brain microvascular endothelial cells (HBMEC), human brain-derived pericytes (BP) and human umbilical arterial smooth muscle cells (HUASMC). Relative mRNA level was normalized to mRNA level of BP. HUVEC versus BP, **P = 0.008* and HUVEC versus HUASMC, **P = 0.007*. (**B**) Western blot probed against Sema3C 48 h after adenoviral transduction of pericytes with GFP and full-length Sema3C vectors. (**C**) Full-length Sema3C and the two isoforms were adenovirally expressed in pericytes and cell lysates probed with anti-Sema3C antibodies. The proteolytic processing of full-length Sema3C and Sema3CΔ13 expression vectors resulted in a short Sema3Cp60 isoform. (**D**) Sema3C-transduced pericytes were treated with 20 µM furin convertase inhibitor Decanoyl-RVKR-CMK (Dec) or 10 µM metalloproteinase inhibitor Batimastat (BB-94) and the conditioned medium was collected after 48 hours. Western blot probed against Sema3C. (**E**) HUVEC spheroids in collagen were treated with conditioned medium with or without VEGF-C (20 ng/mL) or FGF2 (25 ng/mL). After 24 h, the cumulative sprout length per spheroid was quantified. (n= 10 spheroids, mean ± s.e.m, unpaired Student’s *t* test). For VEGF-C stimulation, control versus Sema3C (−VEGF) or (+VEGF), ****P<* 0.0001. For FGF2 treatment*,* control versus Sema3C (−VEGF), ****P<* 0.0001. Control versus Sema3C (+VEGF), ****P*= 0.0002. (**F**) Western blot shows a recombinant human Sema3C-Fc protein and Sema3C in conditioned medium derived from pericytes transduced with full-length Sema3C expression vectors. HUVEC spheroids in a collagen matrix were treated with 500 ng of recombinant Sema3C or control IgG. The representative images show that recombinant Sema3C protein inhibited sprouting angiogenesis. The cumulative sprout length per spheroid was quantified and normalized to IgG-Fc treatment (n = 20 spheroids, mean ± s.e.m; unpaired Student’s *t* test). Control IgG-Fc versus Sema3C-Fc (−VEGF), ***P = 0.001* and control IgG-Fc versus Sema3C-Fc (+VEGF), ***P = 0.009*.

**Supplementary Fig.2**

(**A**) Whole-mount ESM1 and CD31 immunostaining of retinae at P5. Image shows higher amount of ESM1 at the growing vascular front in control IgG-Fc-treated retinae compared to Sema3C-treated retinae. Scale bar, 100 μm. (**B**) Whole-mount Dll4 and CD31 immunostaining of retinae at P5. Image shows the Dll4 expression (arrows) was not affected in control IgG-Fc-treated retinae and Sema3C-treated retinae. Scale bar, 100 μm. Western blot probed against Dll4 in HUVEC after 10 h treatment of Sema3C or control-conditioned medium. Sema3C induced Dll4 protein expression. Sample replicates were labeled by numbers (1-3). Dll4 amount was quantified and normalized to loading control GAPDH (**P* = 0.048, mean ± s.e.m; unpaired Student’s *t* test). (**C**) mRNA expression of tip cell-enriched genes (upper panels) and Notch target genes (lower panels) was evaluated by qPCR in HUVEC after 3 h Sema3C or control conditioned medium treatment. APLN and PDGFB were upregulated by Sema3C (sparse culture: control versus Sema3C, (APLN, **P* = 0.01; PDGFB, **P* = 0.005 and confluent culture: control versus Sema3C (APLN, **P* = 0.02; PDGFB, **P* = 0.03)). Sema3C influences expression of genes that are involved in endothelial cell differentiation in sparse culture (EFNB, **P* = 0.02; HEY1, **P* = 0.04; DLL4, **P* = 0.04) and also in confluent culture (EFNB, **P* = 0.0002; HEY1, **P* = 0.04; DLL4, **P* = 0.03). The increased fold was normalized to control treatment (mean ± s.e.m, n.s., non-significant, paired Student’s *t* test). (**D**) Sema3C induced HUVEC cell repellence in normal culture discs but not when quiescent HUVEC were grown on collagen-coated Xellulin discs. To reach endothelial cell quiescence, HUVEC (2*10^5^ cells) were cultured on Xellulin discs in 1% FCS growth medium for ten days. Confluent monolayers were treated with Sema3C or control-conditioned medium for 30 min and cell morphology was examined by bright field microscopy. Scale bar, 20 μm.

**Supplementary Fig. 3**

(**A**) Serum-starved HUVEC were incubated with conditioned medium for 15 min and cell lysates were subjected to Western blot analysis. The activation of ERK detected by anti-phospho-ERK antibody and normalized to the total ERK protein was not altered. (n = 3; n.s., non-significant using paired Student’s *t* test). (**B**) HUVEC were grown to confluence on gelatin-coated coverslips and treated with VEGF (10 ng/mL) or conditioned medium. After 30 min cells were fixed and the adherens junction proteins VE-cadherin and β-catenin were stained with antibodies. Cell nuclei were stained with DAPI. Arrows indicate gaps between adjacent endothelial cells. Representative of three independent experiments. (**C**) Sema3C induced VE-cadherin internalization. HUVEC were treated with Sema3C, control conditioned medium or VEGF (50 ng/mL). After 30 min cells were lysed, membrane and cytosolic fraction of cell lysates were subjected to Western blot analysis. (**D**) Serum-starved HUVEC were treated with conditioned medium for 30 min. VE-cadherin (arrows at cell junctions) and VEGFR2 were immunostained. Cell nuclei were stained with DAPI. Sema3C disrupted cell junction integrity, which was accompanied with decreased signal intensity of VEGFR2 at cell junctions. VEGFR2 was internalized into the cytosol and detected in endosome-like structures (arrow heads). Scale bar, 20 μm.

**Supplementary Fig. 4**

(**A)** Cell apoptosis was measured in Sema3C- or GFP-expressing pericytes after adenoviral transduction for 48 h. Quantification of caspase-3/-7 activity normalized to control GFP-expressing cells. (n = 3. n.s., non-significant using paired Student’s *t* test). (**B**) Plug sections from the HUVEC/pericyte xenotransplantation assay were immunostained with CD34, cleaved caspase-3 and DAPI. Representative images show co-localized cleaved caspase-3 and CD34-positive cells (arrows). Scale bar, 100 μm. (**C**) Quantification of the number of cleaved caspase-3-stained vessels normalized to total vessel number (n = 8 (control) and n = 7 (Sema3C). (mean ± s.e.m; ****P = 0.0007*, unpaired Student’s *t* test ).

**Supplementary Fig. 5**

(**A**) Sema3C impaired HUVEC adhesion. HUVEC were suspended in 100 µL of Sema3C or control-conditioned medium and plated in 96 wells with different ECM proteins (collagen type I (10 µg/cm^2^), fibronectin (20 µg/mL), 0.2% gelatin and 0.1% (w/v) poly-L-lysine). Non-adherent cells were washed away after 30 min. Remaining cells were fixed and immunostained by DAPI. Adherent cells were counted by cell nuclei and the number was normalized to control treatment. (Three independent experiements, n = 4 wells in each assay, mean ± s.e.m; paired Student’s *t* test. Control versus Sema3C: *collagen type I*, *P* = 0.058; *fibronectin*, **P* = 0.003*; gelatin, *P* = 0.02*; poly-L-lysine, *P* = 0.02). (**B**) HUVEC heptotaxis migration was measured using transwell filters (pore size 8 µm) coated with different ECM proteins after 10 h treatment with Sema3C or control conditioned medium with or without VEGF (25 ng/mL). Transmigrated cells were fixed, immunostained by DAPI (n = 3 wells, mean ± s.e.m; unpaired Student’s *t* test). Collagen type I: control versus Sema3C (-VEGF), **P* = 0.006; control versus Sema3C (+VEGF), **P* = 0.01). (**C**) HUVEC were plated on gelatin-coated coverslips and treated with Sema3C or control medium for 15 min. pY118-paxillin and vinculin proteins were located in focal adhesions (arrows) under control conditions. This was abrogated by Sema3C. Scale bar, 20 μm. Representative of three independent experiments.

**Supplementary Fig. 6**

(**A**) HUVEC were transduced lentiviral shRNA against Nrp-2 or plexinA2 and qPCR analysis was performed after 72 h (NS shRNA versus Nrp-2 shRNA (#1, ***P* = 0.004 and #2, ***P* = 0.001); NS shRNA versus PlexinA2 shRNA (#1, ***P* = 0.002 and #2, ***P* = 0.015)). Cells were fixed and stained with Alexa-Fluor 488-conjugated phalloidin to visualize F-actin. Arrows indicate Sema3C-induced cell membrane ruffling. Scale bar, 20 μm. (**B**) Western blot probed against Nrp-2 and Nrp-1 72 h after lentiviral transduction in HUVEC. Downregulation of Nrp-2 in HUVEC using lentiviral shRNA against Nrp-2. NS shRNA and Nrp-1 were used as control to show specific inhibition. (**C**) HUVEC were transduced shRNA targeting Nrp-2 or plexinA2 and plated on gelatin-coated coverslips. Confluent monolayers were treated with Sema3C, Sema3Cp60 or control conditioned medium for 30 min. Cells were fixed and stained with Alexa-Fluor 488-conjugated phalloidin to visualize F-actin. Arrows indicate Sema3C-induced cell membrane ruffling. Scale bar, 20 μm. (**D**) HUVEC were transfected with siRNA against PlexinD1. Cells were treated with recombinant Sema3C or IgG-Fc protein (500 ng) and fixed 30 min later. F-actin assembly was examined by Alexa-Fluor488 phalloidin. (**E**) HUVEC were lentivirally transduced with shRNA targeting Nrp-1 or Nrp-2 and treated with recombinant Sema3C or IgG-Fc proteins. Depletion of Nrp-1, but not Nrp-2, prevented Sema3C-induced F-actin disassembly. Scale bar, 20 μm. (**F**) HUVEC were treated with Sema3C or Sema3CΔ13 conditioned medium for 10 min, lysed, and Nrp-1 was immunoprecipitated. Bound Sema3C was detected by Western blotting. Secreted Sema3CΔ13 failed to bind Nrp-1.

**Supplementary Fig. 7**

(**A**) Sema3C and its receptors PlexinD1 and Nrp-1 expression in retinae during physiological retinal angiogenesis. Nrp-1 and PlexinD1 were largely expressed in developing retinal vessels in particular at the sprouting front. Sema3C mRNA was not detectable at P4. Scale bar, 20 μm (left panels) and 100 μm (right panels). (**B**) Whole-mount Nrp-1 and CD31 protein immunostaining. Nrp-1 was expressed in pathological pre-retinal tufts (P17, OIR) but not in mature vessels (P17, normal). Same image shown as in Fig. 7B (higher magnification). Scale bar, 100 μm. Whole-mount ISH of retinae (OIR, P16) shows increased mRNA expression of PlexinD1 on pre-retinal vascular tufts. Scale bar, 20 μm. (**C**) Western blot analysis shows Sema3C protein expression in the retinae during development and in the OIR model. Sema3C was probed with anti-Sema3C antibody and mainly detected as p60 isoform.
