# Supplementary material for: Semaphorin-3C signals through Neuropilin-1 and PlexinD1 receptors to inhibit pathological angiogenesis
Source: EMBO Mol Med. 2015 Jul 20;7(10):1267–84. doi: 10.15252/emmm.201404922 (PMC4604683; doi:10.15252/emmm.201404922)
Supplement: Supplementary file 7 [file emmm0007-1267-sd7.pdf]

EMBO Molecular Medicine

Semaphorin-3C signals through Neuropilin-1 and PlexinD1 receptors to inhibit pathological angiogenesis

Wan-Jen Yang, Junhao Hu, Akiyoshi Uemura, Fabian Tetzlaff, Hellmut G. Augustin and Andreas Fischer

Corresponding author: Andreas Fischer, German Cancer Research Center DKFZ

Review timeline:

Submission date:	03 December 2014
Editorial Decision:	13 January 2015
Additional author correspondence :	10 April 2015
Additional Editorial correspondence:	10 April 2015
Revision received:	20 May 2015
Editorial Decision:	12 June 2015
Revision received:	17 June 2015
Accepted:	19 June 2015

Transaction Report:

(Note: With the exception of the correction of typographical or spelling errors that could be a source of ambiguity, letters and reports are not edited. The original formatting of letters and referee reports may not be reflected in this compilation.)

Editor: Roberto Buccione

1st Editorial Decision 13 January 2015

Thank you for the submission of your manuscript to EMBO Molecular Medicine. We have now heard back from the three Reviewers whom we asked to evaluate your manuscript.

We are sorry that it has taken longer than usual to get back to you on your manuscript due to the partial overlap with the Holiday season.

You will see that the three Reviewers are supportive of your work, although they do mention a number of partly overlapping issues. This prevents us from considering publication at this stage. I will not dwell into much detail, as the evaluations are detailed and self-explanatory.

In addition to the various sensible requests for clarification and for better experimental controls to consolidate your findings, two main points of concern emerge. Specifically, more details are asked for concerning the intracellular processing of Semaphorin and more conclusive evidence is necessary to establish PlxD1 and Nrp1 as Semaphorin receptors.

Considered all the above, while publication of the paper cannot be considered at this stage, we would be pleased to consider a revised submission, with the understanding that the Reviewers' concerns must be addressed as outlined above, with additional experimental data where appropriate and that acceptance of the manuscript will entail a second round of review.

Please note that it is EMBO Molecular Medicine policy to allow a single round of revision only and

that, therefore, acceptance or rejection of the manuscript will depend on the completeness of your responses included in the next, final version of the manuscript.

EMBO Molecular Medicine now requires a complete author checklist (http://embomolmed.embopress.org/authorguide#editorial3) to be submitted with all revised manuscripts. Provision of the author checklist is mandatory at revision stage; The checklist is designed to enhance and standardize reporting of key information in research papers and to support reanalysis and repetition of experiments by the community. The list covers key information for figure panels and captions and focuses on statistics, the reporting of reagents, animal models and human subject-derived data, as well as guidance to optimise data accessibility.

As you know, EMBO Molecular Medicine has a "scooping protection" policy, whereby similar findings that are published by others during review or revision are not a criterion for rejection. However, I do ask you to get in touch with us after three months if you have not completed your revision, to update us on the status. Please also contact us as soon as possible if similar work is published elsewhere.

I look forward to seeing a revised form of your manuscript as soon as possible.

***** Reviewer's comments *****

Referee #1 (Comments on Novelty/Model System):

This is a well written manuscript, the experiments and figures are carefully designed and carried out. Furthermore, the issue is novel on several aspects: molecular, angiogenesis and disease-context.

Referee #1 (Remarks):

The authors describe a novel role of Sema3C in regulating VEGF signaling and angiogenesis in a model of pathological retinal angiogenesis, ROP. Sema3C, expressed by cells other than EC, impaired endothelial VEGF signaling and endothelial behavior in vitro and in vivo.

This is a well written manuscript, the experiments and figures are carefully designed and carried out. Furthermore, the issue is novel on several aspects: molecular, angiogenesis and disease-context.

I have few comments:

Is the anti-angiogenic effect of Sema3C specific to VEGF A, or does it inhibit also other growth factor responses, e. g. to VEGF C, FGF, etc?

Does Sema3C inhibit the expression of endothelial tip cell markers in vitro? It is stated that Sema3C reduced the numbers of tip cells in vivo. The question is whether this is a consequence of general EC cell death or also a possible additional suppressive effect on tip cell differentiation.

Which cells of the retina express Sema3C under physiological or pathological conditions? For example, is the remodelling and maturation of retinal vasculature accompanied by an upregulation of Sema3C? Or is there a change of Sema3C expression in the ROP model that might explain the pathology?

Referee #2 (Remarks):

In this paper the authors investigate the activation of endothelial cells and angiogenesis by Sema3C. They identify the putative receptors involved suggesting that Sema3C exerts an anti-angiogenic effect. The experiments are largely well planned. However it is necessary to provide to specific

controls for some experiments, to identify whether pericytes are the physiologic source of Sema3C and which is the most important form of this protein in vivo by discriminating the effect of cleaved or associated-bound Sema3C

Fig 1. The authors demonstrate pericytes carrying the specific adenovector produce the shorter forms of Sema3C. Is this this post-transductional event blocked by furin inhibitor?
Does endogenous Sema3C undergo the same cleavage in physiologic conditions? Do pericytes in normal or altered vasculature (tumor, ROP, retina, skin) express Sema3C?

Fig 2. More controls are required for these experiments. Which is the behaviour of the mixed cultures in which Sema3C-pericyte are transduced by a Sema3C silencing vector? Similarly, which the role of non-cleaved Sema3C? This result could be obtained by transducing the cells with an uncleavable Sema3C mutant. This experiment is important to discriminate the effect between Sema3C-membrane bound and membrane-unbound forms.

Fig 3. The experiments in retina suggest a possible involvement of Notch/Dll4 pathway. Does Sema3C (as positive control the authors can use VEGF) modify the expression of DLL4 in tip cells?

Fig 4. The experiments showing the effect of Sema3C on GFP-Ve-cadherin are interesting. As reported in panel G, I suggest to show the behaviour of endogenous protein in cultured ECs. Furthermore, a fraction of VEGFR2 is known to be associated with Ve-cadherin. Does Sema3C modify VEGFR2 traffic? This result could help understanding the mechanisms triggered by Sema3C.

The in vivo (retina) and in vitro experiments indicate that Sema3c activates apoptosis. One of the mechanism triggering this process is anoikis, which is integrin-dependent. Because class 3 semaphorins inhibit the integrin-mediated adhesive process, the authors have to take into account this aspect providing experiments covering this aspect of Sema function on vascular system This issue has been only partially addressed in Fig 5 and more specific have to be provided (adhesion assay on different substrates, haptotaxis, integrins involved).

Fig 6. By loss-of-functions approaches the authors identify Nrp1 and PlxD1 as Sema3C receptors. These data have to be improved by showing the effect of the specific shRNA not only in term of mRNA but also detecting the amount of proteins. Then to support the specificity of this mechanism, that could be also redundant, and to avoid off-target effects of the shRNAs used, rescue experiments with the specific cDNAs are required.

Fig 3 and 7. By ISH is important to provide the expression of the receptors of Sema3C

Minor point
Fig1S. I suggest to show the blot in HIVEC to detect the amount of Sema3C. In the case of a negative result, I suggest to simply show the data in pericytes

Referee #3 (Remarks):

This manuscript by Yang and coworkers describes the anti-angiogenic activity of Semaphorin 3C, validated in mouse models of physiological and pathological retinal neoangiogenesis.
Sema3C is known to be required for embryonal cardiovascular development; neuropilin-1 and plexin-D1 have been implicated as putative receptors. Other secreted semaphorins, such as Sema3A, 3B, 3E and 3F, were reported to inhibit angiogenesis via receptor complexes formed by members of the neuropilin and plexin families. However, a potential role of Sema3C in angiogenesis has not been characterized.
In this study, Sema3C is shown to inhibit HUVEC-based functional assays in vitro, as well as vessel formation from HUVEC spheroids transplanted subcutaneously in mice. Sema3C impaired physiological postnatal retinal angiogenesis, as well as pathological neoangiogenesis in a model of

post-ischemic retinopathy, however it did not affect mature quiescent vasculature. The authors found that Sema3C suppresses VEGF-mediated endothelial cell survival and migration via both receptors neuropilin-1 and plexin-D1.

The study is novel and relevant to both semaphorin research and angiogenesis field. It is particularly interesting the concept that Sema3C may be capable of selectively inhibiting immature and aberrant vessels without affecting normal vasculature, although the underlying mechanisms should be better elucidated.

Specific issues:
1) In some of the experiments, the authors assayed the activity of recombinant truncated forms of Sema3C, resembling fragments produced by its proteolytic cleavage. However, since Sema3C- 13 lacks the basic-charged stretch normally exposed by furin-cleavage at the C-terminus of the semaphorin, it is unlikely to use the well characterized C-end rule mechanism to interact with Nrp1 (e.g. see Parker et al., J Mol Biol., 2013). At the same time, the data with Sema3Cp60 construct demonstrate that the sema-PSI domain alone is not sufficient to interact with Nrp1 and elicit biological effects. This may suggest a previously unreported functional relevance of the IG domain of secreted semaphorins, which could be discussed. Importantly, in order to elucidate this aspect, the authors could test Sema3C- 13 binding/association with Nrp1 (e.g. as shown for other constructs in Fig. 6D). Note that the PSI domain is located upstream to the internal furin cleavage site, not downstream it; thus Fig. 1A must be appropriately corrected.
2) Figure 2 shows that -in the presence of exogenous Sema3C- few vessels are formed in vivo from HUVEC spheroids, but they are well covered with mural cells. Moreover, Figure 3 shows that Sema3C strongly impaired the development of retinal vasculature at birth, and pruned aberrant vessels during pathological neoangiogenesis, while it did not affect established retinal vasculature in the adult. The authors conclude about a selective effect of Sema3C on immature (mostly naked?) vessels. However, the molecular mechanisms underlying this effect are not well characterized. The authors could investigate differential expression of Sema3C receptors in various stages/conditions in vivo, and/or test Sema3C activity in experimental models characterized by the presence or absence of pericytes beside endothelial cells.
3) The functional requirement for Sema3C receptors, shown in Fig.6 by representative images depicting morphological endothelial cell changes, should be demonstrated by quantifiable assays. For example, the authors could perform endothelial cell migration assays, or sprouting assays (as shown in Fig. 1).

Minor point:
In page 6, line 10, it is stated that Sema3C- 13 is "resembling furin cleavage within c-terminal basic domain"; this is not really accurate, since furin cleavage acts at the C'-end of polybasic amino-acid stretches, and thereby would produces a fragment with C'-tail sequence containing basic-charged amino acid residues -RKSRNRR. It would be correct to say that this recombinant truncated construct lacks the c-terminal basic domain of the semaphorin. Moreover, a protein similar to Sema3C- 13 was previously described by Esselens et al. as being generated by metalloprotease-dependent cleavage.

Additional author correspondence 10 April 2015

Thank you very much for the very positive evaluation of our manuscript. We could successfully address almost all of the reviewers' comments (see below for some details). However, we will still need approximately four weeks to finalize everything. The reason for this is that we had substantial problems to detect endogenous Sema3C expression in mouse tissue by using a large set of commercially available antibodies and RNA in situ. The antibody stainings are not good enough for publication and we do not know anybody in the field who was ever capable of doing this. Although we do not think that this experiment is necessary at all for our manuscript, we now teamed up with two other laboratories (Prof. Gessler, Würzburg, Germany; and Prof Akiyoshi Uemura, Nagoya University, Japan) to do the RNA in situ on retina sections on whole mount. Secondly, we still have to finish a tube formation assay by using a Sema3c mutant that cannot be cleaved by furin. I strongly believe that these experiments will be finished in the next four weeks.

Secondly, I need to inform you that there was a paper published in Cancer Research last week (Mumblat Y, et al. FULL LENGTH SEMAPHORIN-3C IS AN INHIBITOR OF TUMOR LYMPHANGIOGENESIS AND METASTASIS.) showing that Sema3C acts on lymphatic vessels in tumors. There is very little overlap to our study. Only one experiment also shows that Sema3c leads to repellence of HUVEC in the culture dish. We will of course discuss these findings in our revised manuscript and we have further asked Dr. Gera Neufeld, the senior author of this publication, to provide us his non-cleavable Sema3C construct. We will do a tube formation assay and compare this with our constructs. Again, this should be finished within the next four weeks.

This is a short list of the experiments done successfully so far:

1) the Sema3C receptors Nrp1 and PlexinD1 are preferentially expressed in immature blood vessels sprouts during oxygen-induced retinopathy.

2) Sema3C does not affect primary endothelial cells when these are cultured under conditions leading to cell quiescence.

3) Sema3C also interferes with VEGF-C and FGF2-induced angiogenesis.

4) Sema3C slightly interferes with Notch signaling and tip cell formation.

5) There is dynamic Sema3c mRNA expression in retinas during physiological development and oxygen-induced retinopathy.

6) Sema3C cleavage is inhibited by a furin inhibitor.

7) Silencing of Sema3C in pericytes does not alter pericyte functions and angiogenesis of co-cultured endothelial cells.

8) Endogenous VE-Cadherin expression is diminished after Sema3C treatment and there is less VE-Cadherin at the cell membrane

9) Sema3C impairs endothelial cell adhesion to various substrates.

10) specific knockdown of Nrp1, Nrp2 and PlexinD1 is also shown on protein level.

11) Sem3C delta13 does not bind to Nrp1.

12) Membrane ruffling after Sema3C treatment has been analyzed by quantification.

Please do not hesitate to contact me if you have any questions.

Additional Editorial correspondence 10 April 2015

Thank you very much for updating us on the progress of your revision and especially for making us aware of the Cancer Res. manuscript. Besides the fact that of course you would be "covered" by our "scooping protection" policy, I agree that it should not detract much and agree that it should be of course integrated into your revision

Needless to say, no problem with for extra time required. We will make a note of this and in case send you a simple reminder.

In the meanwhile I wish you good luck with the remaining work.

I look forward to reading your revision in due time.

1st Revision - authors' response 20 May 2015

EMM-2014-04922

We thank the Editor and the reviewers for their careful and overall very positive evaluation of our manuscript. We could address all points and this helped to significantly improve the manuscript.

Referee #1

1. Is the anti-angiogenic effect of Sema3C specific to VEGF A, or does it inhibit also other growth factor responses, e.g. to VEGF C, FGF, etc?

This is an interesting question. We performed sprouting assays with HUVEC spheroids in collagen using FGF2 or VEGF-C as growth factors. Indeed, Sema3C inhibited not only VEGF-A-driven angiogenesis, but also antagonized FGF2 and VEGF-C driven angiogenesis (Fig. S1E). This is in line with the literature given that that Nrp-1 binds and regulates the activity of heparin-binding proteins such as VEGF and FGF2 (West et al, JBC, 2005). Also Sema3F, that acts through Nrp2, inhibits FGF2-induced tumor angiogenesis but does not directly interfere with binding of FGF2 to FGFR2 (Kessler et al, Cancer Res, 2004).

2.Does Sema3C inhibit the expression of endothelial tip cell markers in vitro? It is stated that Sema3C reduced the numbers of tip cells in vivo. The question is whether this is a consequence of general EC cell death or also a possible additional suppressive effect on tip cell differentiation.

This is a tricky question since a reduction of tip cell numbers could be due to apoptosis of newly generated tip cells or due to interference with tip cell formation/differentiation. VEGF and Notch signaling drive tip/stalk cell selection in a complex and dynamic manner. We show several data sets suggesting that Sema3C interferes with VEGF signaling, but also with cell survival (Fig. 1C, 1D, 4B and 4D). Now we have performed gene expression analyses of known tip cell "marker genes" (del Toro et al, Blood, 2010) in vitro and also in the neonatal retina. qPCR analyses of HUVEC in both sparse and confluent culture showed decreased expression levels of the tip cell-enriched genes including PDGFB and Apelin (APLN) 3 hours after Sema3C treatment (PDGF-BB to 50%, APLN to 20% of downregulation), whereas other genes including endothelial-specific molecule 1 (ESM1) and Angiopoietin-2 (ANGPT2) were not significantly altered (Fig. S2C). Furthermore, we analyzed

expression of Notch target genes, which are predominantly expressed in stalk cells – with the exception of DLL4. Expression of the Notch target genes ephrin-B2 (EFNB2), DLL4 and HEY1 were increased (130% to 150%) after Sema3C treatment. In addition, we confirmed an increased DLL4 protein expression in HUVEC after Sema3C treatment by Western blot analysis (Fig. S2B).
In order to investigate whether Sema3C also inhibited tip cell marker expression in vivo, we performed intravitreal injection of Sema3C at P3 and harvested the retinae at P5 for immunofluorescence staining. The results showed that ESM1, but surprisingly not Dll4, was strongly downregulated by Sem3C at the sprouting vascular front (Fig. S2A). We do not know why there is a slight difference about which marker gene is regulated by Sema3C in vitro and in vivo. E.g. the published tip cell-enriched genes were derived from isolated retinal endothelial cells without being cultured (Tel Toro et al, Blood, 2010). Nevertheless, based on these results we concluded that Sema3C executes dual effects on tip cells. Sema3C appears to impair endothelial cell survival and tip cell differentiation.

3. Which cells of the retina express Sema3C under physiological or pathological conditions? For example, is the remodelling and maturation of retinal vasculature accompanied by an upregulation of Sema3C? Or is there a change of Sema3C expression in the ROP model that might explain the pathology?

Up to now there is very little knowledge about the expression pattern of Sema3C in mouse, in particular after birth. This may be due to the poor quality for antibodies for IHC or due to the fact that Sema3C is not expressed any more in physiological conditions. We tested several commercial antibodies, which gave no specific results. We and also our collaboration partner Prof. M. Gessler (Biocenter Würzburg, Germany) performed classical mRNA in situ hybridization analyses and again failed to detect specific signals in the retina. Finally, our new cooperation partner Prof. A. Uemura applied a novel mRNA in situ hybridization technique called RNA scope (Gross-Thebing et al, BMC Biol, 2014) that allowed us to examine simultaneously different transcripts in whole mount retinas with high resolution. We examined the expression of Sema3C and PlexinD1 at normal conditions (postnatal day P4) and during pathological conditions (OIR at day P19). The retinae were also immunostained against collagen IV. This revealed that PlexinD1 is expressed in developing retinal vessels in particular at the sprouting front (Fig. S7A), as well as in

pathological retinal tufts (Fig. 7B). Sema3C mRNA was not detectable in the retinas at P4 (Fig. S7A). However, it was expressed during OIR (Fig. 7B). It is clear that the signals came from non-endothelial cells that are very close to the vascular basal membrane. The signal was mainly detected at the vascular tufts that were partly covered by pericytes (NG2 staining) (Fig. 7C).

Furthermore, we did Western blotting against Sema3C in lysates of retinas. This revealed that in OIR conditions Sema3C protein expression is increased. However, we only detected the p60 isoform (Fig. S7C), which we described as inactive. Therefore, it appears that Sema3C is not expressed in the postnatal retina. It can be induced by pathological conditions like in the ROP model, but gets readily inactivated by cleavage. Since the receptors Nrp-1 and PlexinD1 are strongly expressed on pre-retinal tufts and at the sprouting front during angiogenesis (Fig. 7B, 7C, S7A, S7B), it makes much sense to use recombinant cleavage-resistant Sema3C as a therapeutic anti-angiogenic agent delivered locally to the vitreous humor.

Referee #2

Fig 1. The authors demonstrate pericytes carrying the specific adenovector produce the shorter forms of Sema3C. Is this this post-transductional event blocked by furin inhibitor?

Does endogenous Sema3C undergo the same cleavage in physiologic conditions? Do pericytes in normal or altered vasculature (tumor, ROP, retina, skin) express Sema3C?

We added the specific convertase inhibitor Decanoyl-RVKR-CMK (Dec) to pericytes that were transduced with Sema3C expression vectors. Western blot analysis of conditioned medium showed that the convertase inhibitor blocked the cleavage of Sema3C into the p60 isoform (Fig. S1D).

Sema3C gets also cleaved by metalloproteinases like ADAMTS1, at least in tumor cells (Esselens et al, JBC, 2010). However, the metalloproteinase inhibitor Batimastat (BB-94) had no effect on the processing of Sema3C into the p60 isoform (Fig. S1D). This indicates that in pericyte culture the proteolytic processing of Sema3C into the p60 isoform is mainly executed by furin or furin-like protein covertases.

To examine if endogenous Sema3C undergoes proteolytic processing in physiological conditions, we performed Western blot analysis using the whole retina

lysates at different developmental stages and with or without hyperoxia treatment (ROP model). Sema3C was only detected as the cleaved p60 isoform (Fig. S7C). Additionally, mRNA in situ analyses revealed that Sema3C is not expressed in the postnatal retina, but in peri-endothelial cells during oxygen-induced retinopathy (RNA scope, Fig. 7B). These analyses also demonstrated that the receptors Nrp-1 and PlexinD1 are strongly expressed at the sprouting front and in pre-retinal tufts during OIR (Fig. 7B, 7C). Therefore, it appears that the pathophysiologic response to vascular damage in terms of Sema3C expression is weak. Our approach of local Sema3C administration overcomes this limit and leads to death of immature vessels.

Fig 2. More controls are required for these experiments. Which is the behaviour of the mixed cultures in which Sema3C-pericyte are transduced by a Sema3C silencing vector? Similarly, which the role of non-cleaved Sema3C? This result could be obtained by transducing the cells with an uncleavable Sema3C mutant. This experiment is important to discriminate the effect between Sema3C-membrane bound and membrane-unbound forms.

First of all we would like to emphasize that this research project was not aimed at understanding the physiological function of Sema3C. In that case we would have employed Sema3-/- mice and performed siRNA silencing in cell culture. Nevertheless, we performed a tube formation assay with HUVEC that were cocultured with pericytes silenced for Sema3C expression. The data showed that there was no difference when Sema3C was silenced or not (see figure below). This may be due to the fact that Sema3C is expressed at low levels in normal culture conditions or due to redundancy with other Semaphorins. However, we also made the experience in the past, that this assay is not very suitable to show increased angiogenesis. Since we provide no additional data about Sema3C silencing we propose to not include this figure into the manuscript.

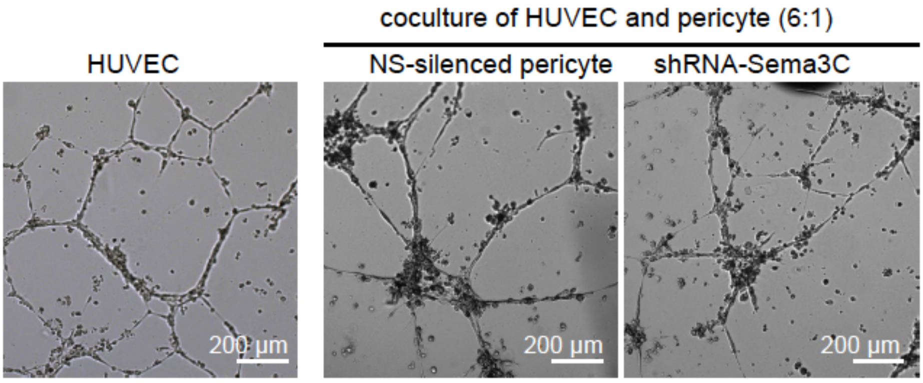

We agree with the reviewer that it is important to investigate the role non-cleavable Sema3C. First of all, the recombinant Sema3C protein we used in many experiments cannot be processed into the p60 isoform since it carries mutations preventing this (Fig. S1G). Our in vitro and in vivo data clearly demonstrated that this "non-cleaved" Sema3C has strong anti-angiogenic functions (Fig.3, 7D, S1G, S5D, S5E). Therefore we already performed this experiment.

Furthermore, we generated a construct (Sema3C-DM) that contains point mutations in the furin consensus motifs. The first KRRSRR sequence was mutated to KARSRA and the c-terminal RKSRNRR was changed to RKSANAA. This protein also inhibited tube formation. While this manuscript was in revision Dr. Gera Neufeld published a paper about the role of a non-cleavable Sema3C in Cancer Res (Mumblat et al, 2015). The cleavage-resistant mutant called Sema3C-FR in this paper has a deletion at the c-terminal basic domain but is still secreted. This mutant could inhibit lymphangiogenesis in tumors. Since this issue is extensively addressed and answered by the Mumblat et al paper, we feel that it would not be suitable to address this issue in our manuscript for a second time.

Fig 3. The experiments in retina suggest a possible involvement of Notch/Dll4 pathway. Does Sema3C (as positive control the authors can use VEGF) modify the expression of DLL4 in tip cells?

We have performed gene expression analyses of known tip cell "marker genes" in vitro. qPCR analyses of HUVEC in both sparse and confluent culture showed increased expression levels of Dll4 3 hours after Sema3C treatment. This was also confirmed by Western blot analysis showing an increased Dll4 protein expression (Fig. S2B). Interestingly, other Notch target genes ephrin-B2 (EFNB2), and HEY1 were also increased (130% to 150%) after Sema3C treatment (Fig. S2C). This indicates that Sema3C would promote the stalk cell phenotype. This was supported by decreased expression levels of the tip cell-enriched genes including PDGFB and Apelin (APLN) after Sema3C treatment. In addition, we confirmed an increased DLL4 protein expression in HUVEC after Sema3C treatment by Western blot analysis (Fig. S2B).

In order to investigate whether Sema3C also inhibited tip cell marker expression in vivo, we performed intravitreal injection of Sema3C at P3 and harvested the retinas at P5 for immunofluorescence staining. The results showed that a tip cell-enriched

protein ESM1, but not Dll4, was strongly downregulated by Sem3C at the sprouting vascular front (Fig. S2A, S2B). Surprisingly, there is some discrepancy between in vitro and in vivo analyses. Nevertheless, based on these results we concluded that Sema3C executes dual effects on tip cells. Sema3C impaired endothelial cell survival and tip cell differentiation.

Fig 4. The experiments showing the effect of Sema3C on GFP-Ve-cadherin are interesting. As reported in panel G, I suggest to show the behaviour of endogenous protein in cultured ECs.

We showed immunofluorescence staining of endogenous VE-cadherin in Fig. S3B. Sema3C caused instability of cell junctions as shown by discontinuous alignment of VE-cadherin. We also performed Western blot analysis of HUVEC treated with Sema3C for 30 minutes and confirmed that the membrane fraction of VE-cadherin was decreased 30 minutes after Sema3C treatment (Fig. S3C).

Furthermore, a fraction of VEGFR2 is known to be associated with Ve-cadherin. Does Sema3C modify VEGFR2 traffic? This result could help understanding the mechanisms triggered by Sema3C.

The co-immunostaining of VEGFR2 and VE-cadherin showed that VEGFR2 is localized at junctions in cells and partly in endosome-like vesicles in cells treated with control medium. Treatment with Sema3C caused an accumulation of VEGFR2 in the cytoplasm and a pronounced reduction at the membrane (Fig. S3D). Thus, it is possible that Sema3C interferes with VEGF signaling via inhibiting VEGFR2 location at cell junctions where VEGFR2 interacts with VE-Cadherin to facilitate AKT activation.

The in vivo (retina) and in vitro experiments indicate that Sema3c activates apoptosis. One of the mechanism triggering this process is anoikis, which is integrin-dependent. Because class 3 semaphorins inhibit the integrin-mediated adhesive process, the authors have to take into account this aspect providing experiments covering this aspect of Sema function on vascular system This issue has been only partially addressed in Fig 5 and more specific have to be

provided (adhesion assay on different substrates, haptotaxis, integrins involved).

We fully agree with the reviewer that loss of cell-cell and cell-matrix contacts with drives endothelial cells into anoikis. This has been added to the manuscript. Furthermore, we performed cell adhesion experiments using different substrates. The data showed that Sema3C inhibited HUVEC adhesion to different ECM proteins but also poly-L-lysine, indicating that Sema3C suppressed endothelial cell adhesion in both integrin-dependent and integrin-independent manners (Fig. S5A).
We also performed haptotaxis assays by using transwell filters (pore size 8 µm) coated with different ECM proteins. HUVEC migration was measured 10 hours after treatment with Sema3C or control conditioned medium. The data showed that Sema3C strongly inhibited HUVEC chemotactic migration on collagen type I-coated membranes, but also on other coating substrates (Fig. S5B).

Fig 6. By loss-of-functions approaches the authors identify Nrp1 and PlxD1 as Sema3C receptors. These data have to be improved by showing the effect of the specific shRNA not only in term of mRNA but also detecting the amount of proteins. Then to support the specificity of this mechanism, that could be also redundant, and to avoid off-target effects of the shRNAs used, rescue experiments with the specific cDNAs are required.

We performed Western blot analysis of HUVEC that were transduced siRNA or shRNA against PlexinD1, Nrp-1 or Nrp-2. The data showed sufficient reduction of protein expression (Fig. 6A, 6C, S6B). Secondly, we showed that shRNA against Nrp-1 does not affect Nrp2 expression, and shRNA against Nrp2 had no influence on Nrp-1 expression (Fig. S6B).
Thirdly, we also generated a human FLAG-tagged Nrp-1 expression vector in which the cDNA sequence was slightly changed so that the shRNA binding was prevented. This vector could rescue the shRNA effects. However, the outcome varied quite a bit every time we repeated the experiment. Since we cannot rule out that these minor changes lead to unpredictable side-effects, and since we achieve artificial overexpression of Nrp-1, with unknown consequences, we did not include this into the revised manuscript.

Fig 3 and 7. By ISH is important to provide the expression of the receptors of Sema3C

We now show mRNA expression of PlexinD1 and Sema3C in the retina at P4 and during OIR at P19. PlexinD1 is strongly expressed at the sprouting front and in pre-retinal tufts (Fig. 7B, S7A). Nrp-1 protein expression could be analyzed by immunofluorescence staining. Nrp-1 is highly expressed at the sprouting front in retinal vessels at day P4 (Fig. S7A). Its expression is much weaker in mature retinal vessels at P17 (Fig. S7B). In the OIR model Nrp-1 was abundantly expressed on pre-retinal tufts (Fig. 7C). These data strengthen our hypothesis that Sema3C selectively affects immature blood vessels.

Minor point

Fig1S. I suggest to show the blot in HUVEC to detect the amount of Sema3C. In the case of a negative result, I suggest to simply show the data in pericytes

We performed Western blot analysis and now show Sema3C expression in HUVEC (none) and brain-derived pericytes (weak).

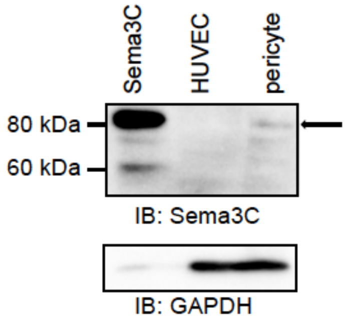

Referee #3 (Remarks):

1) In some of the experiments, the authors assayed the activity of recombinant truncated forms of Sema3C, resembling fragments produced by its proteolytic cleavage. However, since Sema3C-Δ13 lacks the basic-charged stretch normally exposed by furin-cleavage at the C-terminus of the semaphorin, it is unlikely to use the well characterized C-end rule mechanism to interact with

Nrp1 (e.g. see Parker et al., J Mol Biol., 2013). At the same time, the data with Sema3Cp60 construct demonstrate that the sema-PSI domain alone is not sufficient to interact with Nrp1 and elicit biological effects. This may suggest a previously unreported functional relevance of the IG domain of secreted semaphorins, which could be discussed.
Importantly, in order to elucidate this aspect, the authors could test Sema3C-Δ13 binding/association with Nrp1 (e.g. as shown for other constructs in Fig. 6D). Note that the PSI domain is located upstream to the internal furin cleavage site, not downstream it; thus Fig. 1A must be appropriately corrected.

The reviewer raised an interesting point. Although it was not the aim of this work to shed more light into the complicated issue of Sema3 proteolytic processing and elucidation of the isoforms' biology, we performed additional experiments in this direction. First, of all we changed Fig1A accordingly. Secondly, we treated pericytes with the convertase inhibitor Decanoyl-RVKR-CMK (Dec). Western blot analysis of conditioned medium showed that the convertase inhibitor blocked the cleavage of Sema3C into the p60 isoform (Fig. S1D). Sema3C gets also cleaved by metalloproteinases like ADAMTS1, at least in tumor cells (Esselens et al, 2010). The metalloproteinase inhibitor Batimastat (BB-94) had no effect on the processing of Sema3C into the p60 isoform as expected (Fig. S1D).
Thirdly, we examined the expression of endogenous Sema3C RNAscope and classical mRNA in situ. This revealed that Sema3C is most likely not expressed in the retina at P4 but its expression gets induced in the OIR model in peri-endothelial cells (Fig. 7B and S7A). Forth, we performed Western blot analysis using the whole retina lysates at different developmental stages and with or without hyperoxia treatment (OIR model). Very little Sema3C protein was detected and importantly, only the p60 isoform (Fig. S7C).
Next we addressed the delta13 isoform. Indeed this isoform lacks the basic stretch that would allow Nrp-1 interaction via the C-end rule mechanism. We performed co-immunoprecipitation of endogenous Nrp-1 and Sema3C-Δ13. While Sema3C-conditioned medium acted as a positive control for Nrp-1 binding, Sema3C-Δ13 interacted not with Nrp-1 (Fig. S5G). This is true at least for these experimental conditions and supports the general concept of the C-end rule as predicted by the reviewer. Nevertheless, this delta13 isoform was clearly active in our hands (Fig. 1B, 1C). It is obviously beyond the scope of this manuscript to further elucidate the molecular mechanisms of the delta13 isoform and the other Sema3C domains e.g.

the Ig-domain, that was shown to bind Nrp-1 and Nrp-2 (Neuron. 1998 Dec;21(6):1283-90).
Interestingly, a paper published recently by Dr. Gera Neufeld (Mumblat et al, Cancer Res, 2015) shows that a cleavage-resistant Sema3C mutant with a deletion for Furin and/or ADAMTS1 cleavage at the c-terminus is biologically active.

2) Figure 2 shows that -in the presence of exogenous Sema3C- few vessels are formed in vivo from HUVEC spheroids, but they are well covered with mural cells. Moreover, Figure 3 shows that Sema3C strongly impaired the development of retinal vasculature at birth, and pruned aberrant vessels during pathological neoangiogenesis, while it did not affect established retinal vasculature in the adult. The authors conclude about a selective effect of Sema3C on immature (mostly naked?) vessels. However, the molecular mechanisms underlying this effect are not well characterized. The authors could investigate differential expression of Sema3C receptors in various stages/conditions in vivo, and/or test Sema3C activity in experimental models characterized by the presence or absence of pericytes beside endothelial cells.

We now show mRNA expression of PlexinD1 and Sema3C in the retina at P4 and during OIR at P19. PlexinD1 is strongly expressed at the sprouting front and in pre-retinal tufts (Fig. 7B and S7A). Nrp-1 protein expression could be analyzed by immunofluorescence staining. Nrp-1 is highly expressed at the sprouting front in retinal vessels at day P4 (Fig. S7A). Its expression is much weaker in mature retinal vessels at P17 (Fig. S7B). In the OIR model Nrp-1 was abundantly expressed on pre-retinal tufts (Fig. 7B). These data strengthen our hypothesis that Sema3C selectively affects immature blood vessels since its receptors are strongly expressed on endothelial cells of immature vessels.
To support this hypothesis also in cellular models, we performed two *in vitro* assays. First, we used the spheroid-based sprouting angiogenesis assay with mixed HUVEC/ pericytes (5:1 ratio) spheroids. However, we observed that the endothelial cells were not sensitive to VEGF stimulation and did not sprout at all (only pericytes formed tube-like structures). Therefore, we employed a novel culture system that allows long-term culture (more than a year) of HUVEC in quiescent conditions. Importantly, the cells do not loose their endothelial characteristics and they start proliferation once transferred back to normal cell culture conditions. Since the method has not yet been

published, we provide Reviewers and Editors with CONFIDENTIAL material (removed by the editor) provided by the company (Bioregeneration, Munich, Germany).
Xellulin, which is a hydrogel scaffold, was coated with collagen und HUVEC were added to form a confluent monolayer. This induces cell quiescence after just a few days of culture (10 days). We compared the morphology of HUVEC monolayer that were grown in normal culture condition and on Xellulin discs after the treatment with Sema3C or control conditioned medium. Sema3C induced HUVEC repellence in normal cell culture but not on Xellulin (Fig. S2D). These data further prove our hypothesis that quiescent endothelial cells are protected from Sema3C-mediated effects.
Please also note: in the xenotransplantation assay it may well be that not all endothelial cells are close enough to the co-grafted pericytes to receive high levels of Sema3C. Therefore it is not surprising that they form intact vessels that recruit peri-endothelial cells.

3) The functional requirement for Sema3C receptors, shown in Fig.6 by representative images depicting morphological endothelial cell changes, should be demonstrated by quantifiable assays. For example, the authors could perform endothelial cell migration assays, or sprouting assays (as shown in Fig. 1).

We have quantified the number of cells with membrane ruffling (Fig 6B and 6C). As an alternative there was only the spheroid-based sprouting assay. However, Nrp-1 is an important co-receptor of VEGFR2 and its silencing would already impair sprouting. Indeed, we observed that knockdown endogenous Nrp-1 in HUVEC caused the reduction of endothelial sprouting angiogenesis not only under basal levels but also with VEGF stimulation (see figure below). Therefore, it would be impossible to evaluate if Sema3C anti-angiogenic effects can be rescued by Nrp-1 knockdown.

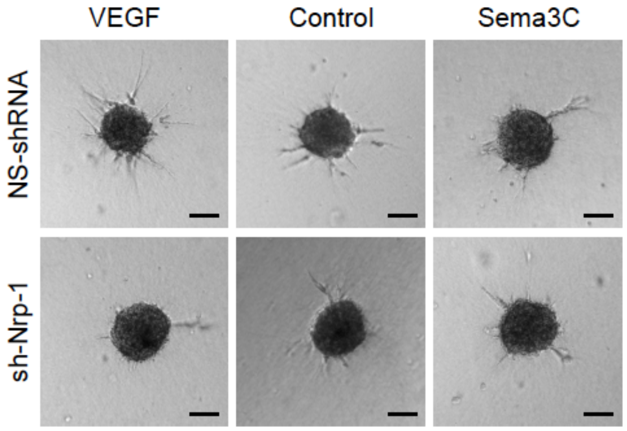

Minor point: In page 6, line 10, it is stated that Sema3C-Δ13 is "resembling furin cleavage within c-terminal basic domain"; this is not really accurate, since furin cleavage acts at the C'-end of polybasic amino-acid stretches, and thereby would produces a fragment with C'-tail sequence containing basic-charged amino acid residues -RKSRNRR. It would be correct to say that this recombinant truncated construct lacks the c-terminal basic domain of the semaphorin. Moreover, a protein similar to Sema3C-Δ13 was previously described by Esselens et al. as being generated by metalloprotease-dependent cleavage.

We fully agree. The sentences have been corrected accordingly. Indeed, the isoform Sema3C-Δ13 was generated according to the publication by Esselens et al., and resembles metalloprotease-dependent protein cleavage. As stated above (comments to reviewer #2) it does not obey the C-end rule as expected.

<u>Cited literature in the rebuttal letter:</u>

del Toro R, Prahst C, Mathivet T, Siegfried G, Kaminker JS, Larrivee B, Breant C, Duarte A, Takakura N, Fukamizu A et al (2010) Identification and functional analysis of endothelial tip cell-enriched genes. Blood 116**:** 4025-4033

Esselens C, Malapeira J, Colome N, Casal C, Rodriguez-Manzaneque JC, Canals F, Arribas J (2010) The cleavage of semaphorin 3C induced by ADAMTS1 promotes cell migration. The Journal of biological chemistry 285**:** 2463-2473

Gross-Thebing T, Paksa A, Raz E (2014) Simultaneous high-resolution detection of multiple transcripts combined with localization of proteins in whole-mount embryos. BMC biology 12**:** 55

Kessler O, Shraga-Heled N, Lange T, Gutmann-Raviv N, Sabo E, Baruch L, Machluf M, Neufeld G (2004) Semaphorin-3F is an inhibitor of tumor angiogenesis. Cancer research 64**:** 1008-1015

Mumblat Y, Kessler O, Ilan N, Neufeld G (2015) Full Length Semaphorin-3c Is an Inhibitor of Tumor Lymphangiogenesis and Metastasis. Cancer research

West DC, Rees CG, Duchesne L, Patey SJ, Terry CJ, Turnbull JE, Delehedde M, Heegaard CW, Allain F, Vanpouille C et al (2005) Interactions of multiple heparin binding growth factors with neuropilin-1 and potentiation of the activity of fibroblast growth factor-2. The Journal of biological chemistry 280**:** 13457-13464

2nd Editorial Decision 12 June 2015

Thank you for the submission of your revised manuscript to EMBO Molecular Medicine. We have now received the enclosed reports from the referees that were asked to re-assess it. As you will see the reviewers are now globally supportive and I am pleased to inform you that we will be able to accept your manuscript pending the following final amendments:

1) While performing our pre-publishing quality control and image screening routines, we noticed that the box highlighting the magnified area displayed in the inset of Fig. 4F (Sema3C-Fc) is not correctly positioned (as per the attached screenshot). Please provide a corrected figure file.

2) The manuscript must include a statement in the Materials and Methods identifying the institutional and/or licensing committee approving the experiments, including any relevant details (like how many animals were used, of which gender, at what age, which strains, if genetically modified, on which background, housing details, etc). We encourage authors to follow the ARRIVE guidelines for reporting studies involving animals. Please see the EQUATOR website for details: http://www.equator-network.org/reporting-guidelines/improving-bioscience-research-reporting-the-arrive-guidelines-for-reporting-animal-research/. Please make sure that all the above details are reported. This information should have been included in the checklist I had asked you to submit with your revision and which you did not provide.

3) We are now encouraging the publication of source data, particularly for electrophoretic gels and blots, with the aim of making primary data more accessible and transparent to the reader. Would you be willing to provide a PDF file per figure that contains the original, uncropped and unprocessed scans of all or at least the key gels used in the manuscript? The PDF files should be labeled with the appropriate figure/panel number, and should have molecular weight markers; further annotation may be useful but is not essential. The PDF files will be published online with the article as supplementary "Source Data" files. If you have any questions regarding this just contact me.

Please submit your revised manuscript within two weeks. I look forward to seeing a revised form of your manuscript as soon as possible.

***** Reviewer's comments *****

Referee #1 (Remarks):

My concerns have been properly addressed by new experiments. I have no further comments and find the manuscript suitable for publication.

Referee #2 (Comments on Novelty/Model System):

The authors use wide and adequate technologies to experimentally face their hypotheses

Referee #2 (Remarks):

I have not further comments

Referee #3 (Remarks):

Not all of the issued raised in my review have been addressed; however, the revised manuscript by Yang and colleagues is significantly improved compared to the initial version, and provides relevant

information and compelling evidence about a previously unknown inhibitor activity of Sema3C in physiological and pathological angiogenesis.

2nd Revision - authors' response 17 June 2015

we would like to thank you and the three referees for the positive evaluation of our revised manuscript. According to your comments we made the following changes:

1) Fig. 4F was corrected and the inset replaced. We thank you very much for this very careful quality check.

2) We found another mistake in Fig. 6 where one cropped blot was presented upside down. We have corrected this and provide a revised Fig. 6.

3) We corrected the figure legend 1. Here there was a mix up for p values and the number of mice. The figure is correct and the changes do not interfere with interpretation of the data etc.

4) We included a statement about the licensing committee for animal experiments. We included the sex of the mice used for experiments. Additionally, please find all these details in the Checklist. I was pretty sure that I had uploaded this document last time. Nevertheless, I will upload it now as an Excel file.

5) We provide source data for Figures 4, 5 and 6 as single PDF files.

Please let me know if you need anything else.
