# Supplementary figures and images for: Semaphorin-3C signals through Neuropilin-1 and PlexinD1 receptors to inhibit pathological angiogenesis
Source: EMBO Mol Med. 2015 Jul 20;7(10):1267–84. doi: 10.15252/emmm.201404922 (PMC4604683; doi:10.15252/emmm.201404922)

Source Data for Fig. 4C

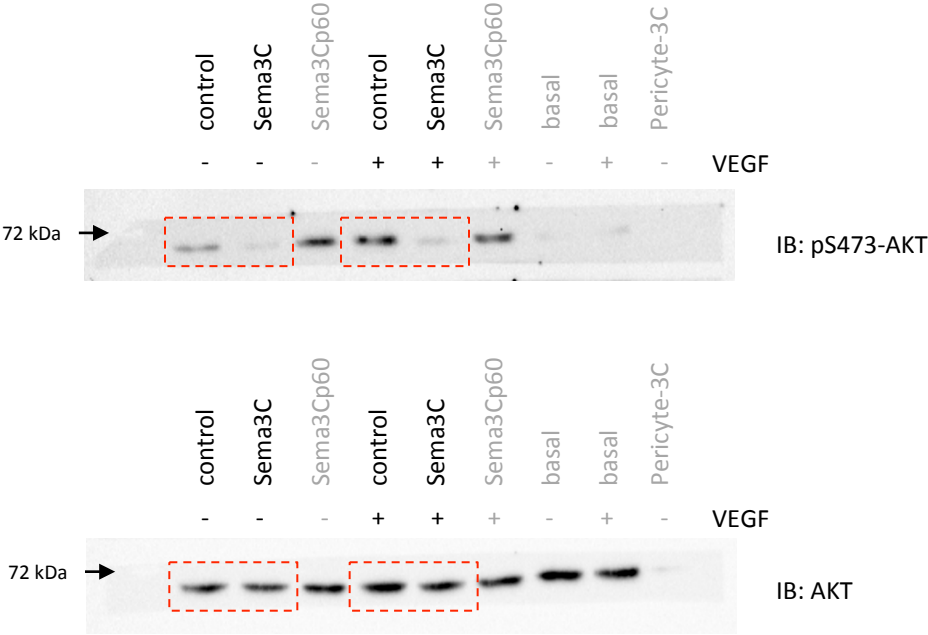

Supplement: Supplementary file 8 [file emmm0007-1267-sd8.pdf]

Source Data for Fig. 5D and 5E

Fig. 5D

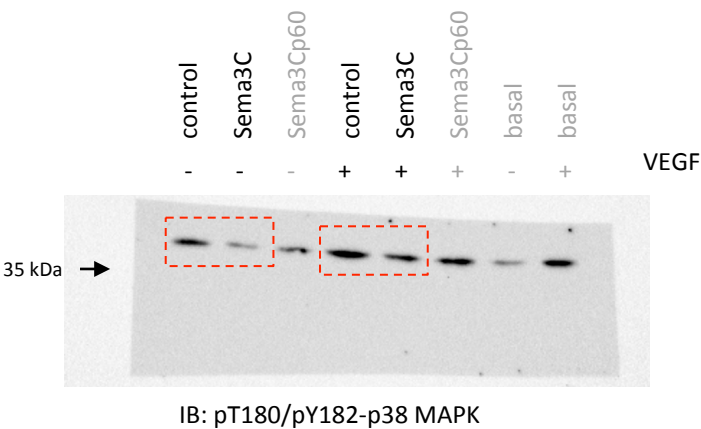

Fig. 5E

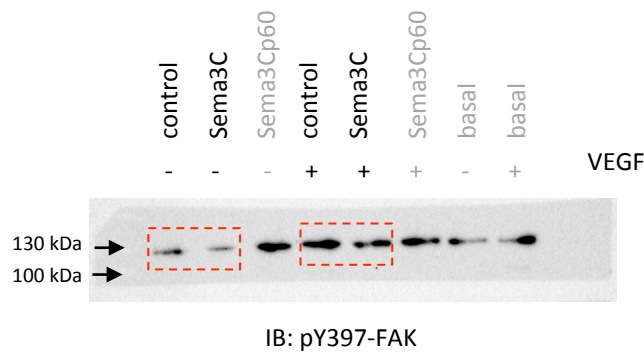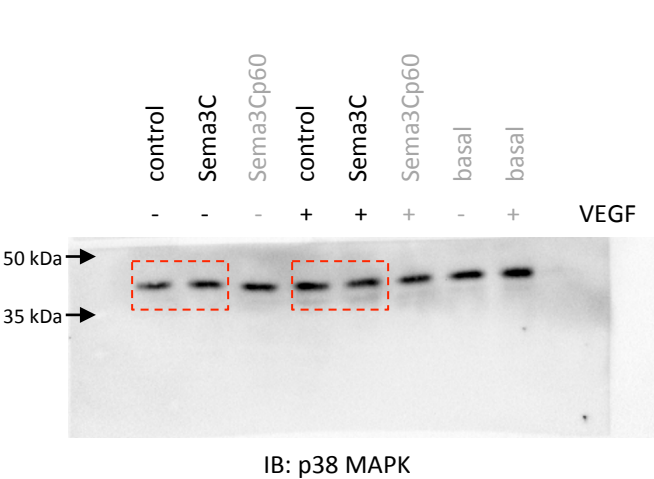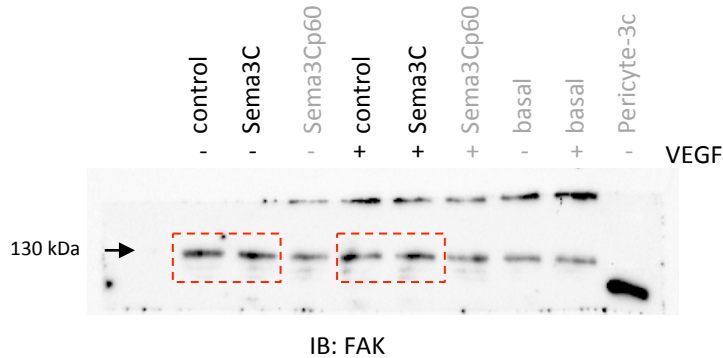

Supplement: Supplementary file 9 [file emmm0007-1267-sd9.pdf]

Source Data for Fig. 6A, 6C and 6E

Fig. 6A

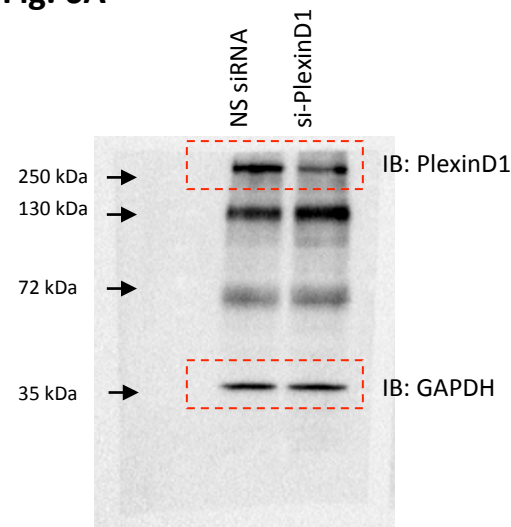

Fig. 6C

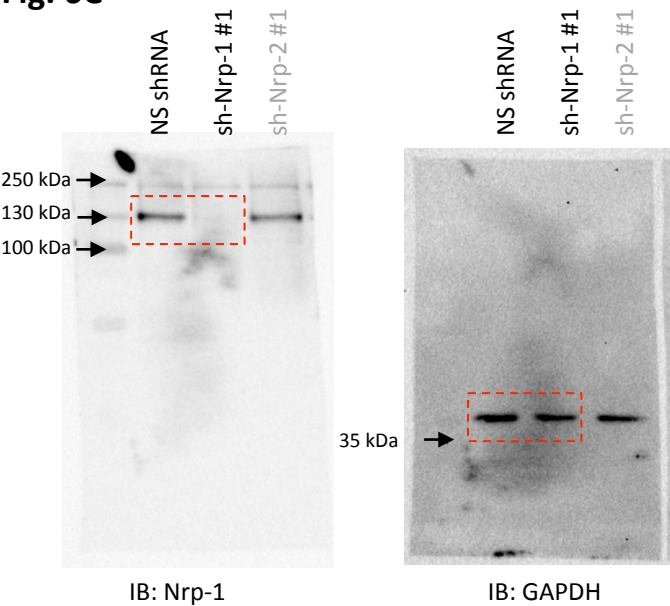

Fig. 6E

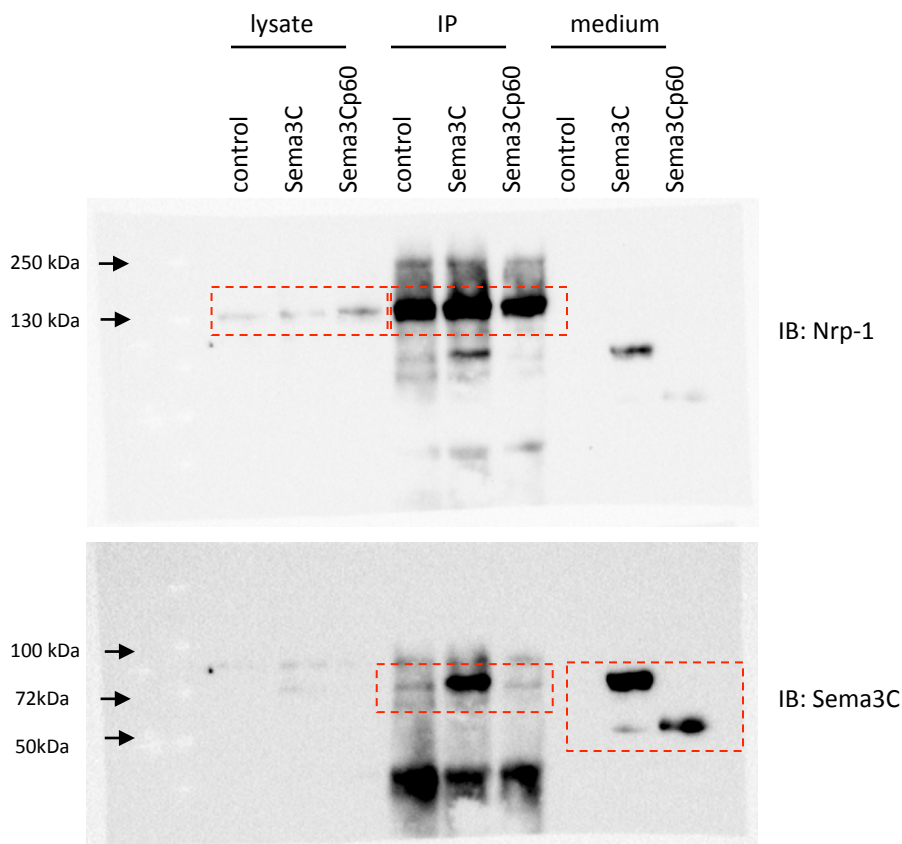

Supplement: Supplementary file 10 [file emmm0007-1267-sd10.pdf]
